# Real-World Evidence of the Effectiveness and Safety of Ustekinumab for the Treatment of Crohn’s Disease: Systematic Review and Meta-Analysis of Observational Studies

**DOI:** 10.3390/jcm11144202

**Published:** 2022-07-20

**Authors:** Cristina Rubín de Célix, María Chaparro, Javier P. Gisbert

**Affiliations:** Gastroenterology Department, Hospital Universitario de La Princesa, Instituto de Investigación Sanitaria Princesa (IIS-Princesa), Universidad Autónoma de Madrid (UAM), Centro de Investigación Biomédica en Red de Enfermedades Hepáticas y Digestivas (CIBEREHD), 28006 Madrid, Spain; mariachs2005@gmail.com (M.C.); javier.p.gisbert@gmail.com (J.P.G.)

**Keywords:** ustekinumab, Crohn’s disease, effectiveness, safety, real-world evidence

## Abstract

(1) Background: Evidence on the outcomes of ustekinumab treatment in real-world Crohn’s disease (CD) patients is needed. Our aim was to evaluate the effectiveness and safety of ustekinumab in CD, reported by observational studies. (2) Methods: bibliographical searches were performed (PubMed, EMBASE). Selection: observational studies assessing the effectiveness and safety of ustekinumab in CD. Exclusion criteria: studies using ustekinumab as a prophylaxis for postoperative recurrence or perianal disease. Data synthesis: effectiveness by intention-to-treat (random-effects model). Data were stratified by study design, population included, administered dose, and prior biologic exposure. (3) Results: A total of 63 studies (8529 patients) were included. Response was achieved in 60% (95% CI, 54–67%) in the short term (8–14 weeks); 64% (57–71%) in the medium term (16–24 weeks); and 64% (52–74%) in the long term (48–52 weeks). Remission was achieved in 37% (28–46%) in the short term; 42% (36–49%) in the medium term; and 45% (37–53%) in the long term. The endoscopic remission rate was 33% (25–40%) in the long term. Eighteen percent of patients lost response during follow-up. Nearly one-third of the patients needed dose optimisation, and in 59% of them it was effective. Twenty-five percent of patients developed adverse events, leading to treatment withdrawal in seven percent of the cases. (4) Conclusions: Ustekinumab is an effective and safe therapy in real-world refractory CD patients. Dose optimisation is frequently required, being effective in a high percentage of cases.

## 1. Introduction

Inflammatory bowel disease (IBD) comprises a series of chronic disorders of unknown cause affecting the gastrointestinal tract, and it is associated with a complex immune response. Treatment options for IBD are rapidly expanding because the currently available treatments are still ineffective in many patients [1,2,3,4].

Ustekinumab is a fully human monoclonal IL-12/23 p40 antibody. IL-12 and IL-23 play a key role in the inflammatory cascade in Crohn’s disease (CD). According to the summary of product characteristics, ustekinumab is approved for the treatment of patients with moderate-to-severe CD who have had an inadequate response with, lost response to, or were intolerant to either conventional therapy or to anti-tumour necrosis factor (anti-TNF), or have medical contraindications to these therapies [5].

The efficacy of ustekinumab has been shown in the UNITI-1, UNITI-2 (induction) and IM-UNITI (maintenance) clinical trials [6,7,8]. As with other biological treatments, efficacy was greater in patients naïve to anti-TNF drugs (UNITI-2) than in previous non-responders to such drugs (UNITI-1). The safety profile of the drug is favourable. Nevertheless, randomised clinical trials may not represent the real-world IBD population because an important proportion of IBD patients do not meet their strict inclusion criteria [9,10].

In this scenario, in the last few years, observational studies reporting the effectiveness and the safety of ustekinumab for CD have been conducted. However, the published studies are scarce, with few patients mainly included in unicentric cohorts. Therefore, the effectiveness and safety of ustekinumab are not yet clear. The aim of this study was to evaluate, through a systematic review and meta-analysis, the effectiveness and safety of ustekinumab in the treatment of CD reported by observational studies.

## 2. Materials and Methods

### 2.1. Literature Search and Study Selection

Bibliographic searches were performed in PubMed and EMBASE up to December 2021. The search strategy (with corresponding keywords in all fields) was: (“inflammatory bowel disease” OR “crohn’s disease”) AND ustekinumab. Additional hand searches were performed by cross-referencing eligible studies in order to identify further relevant publications. We also included conference proceedings of the last five years from Digestive Diseases Week (DDW), United European Gastroenterology Week (UEGW), the European Crohn’s and Colitis Organisation (ECCO), and the World Gastroenterology Organisation (WGO). Abstracts were screened to discard duplicates, and when the literature search yielded two or more studies by the same author assessing the same populations, only the most recent one was chosen, irrespective of the time interval, as it was assumed that the last one published would include the most comprehensive and complete data. The corresponding authors of the studies without sufficient data were contacted for additional information. The process of study selection is depicted in a flow diagram following the PRISMA statement [11]. The present systematic review was registered in PROSPERO (CRD42021273274).

Two reviewers (CR and MC) selected the articles, first by title and abstract and then by full-text review and following the selection criteria. Any discrepancy during the selection of references was solved by consensus with a third reviewer (JPG).

### 2.2. Selection Criteria

Prospective and retrospective studies assessing the effectiveness or the safety of ustekinumab in CD were selected for inclusion. There were no language restrictions, and studies focused on paediatric patients could be included. Articles in which ustekinumab had been prescribed exclusively as prophylaxis for postoperative recurrence in CD or for perianal CD were excluded. Systematic or narrative reviews and clinical trials were excluded from this systematic review.

### 2.3. Data Extraction

A predefined data-extraction form was used to collect the data. The variables recorded were: year of publication; study design (prospective or retrospective); age of the study population (adults or children); sample size; previous biologic exposure (naïve or non-naïve); use of concomitant immunomodulator therapy; administered dose of ustekinumab; ustekinumab as induction or maintenance therapy; length of follow-up (in months); outcome measures (clinical and endoscopic response, clinical and endoscopic remission, and corticosteroid-free clinical remission); and predictors of response (if any). Outcome measures were reported in the short term (8–14 weeks), in the medium term (16–24 weeks), and in the long term: 48–52 weeks, where available. We also collected the need for dose optimisation, median time of initiation of therapy intensification, and effectiveness of dose optimisation. Dose escalation was defined as a shortening of the administration interval from every 12 weeks to every 8 weeks; dose intensification was defined as a shortening of the ustekinumab administration interval to less than 8 weeks (every 4 or 6 weeks) or administration of a reinduction IV dose of ustekinumab. Adverse events (AEs) associated with ustekinumab treatment were also recorded (including AEs that required ustekinumab discontinuation and serious AEs related to ustekinumab). We defined serious AEs following the criteria of the Food and Drug Administration (FDA): the need for hospitalisation, disability or permanent damage, life-threatening, required intervention to prevent permanent impairment or damage, congenital anomaly/birth defect, or death.

### 2.4. Quality Assessment

To assess the quality of the observational studies (only the full-text ones) we used the “Newcastle-Ottawa Scale” (NOS), which is considered the most reliable method for outcome assessment [12]. The evaluated items of NOS are detailed in Figure S1.

### 2.5. Outcome Measures

#### 2.5.1. Primary Outcomes

1. Clinical and endoscopic response and remission in CD patients treated with ustekinumab in the short, medium, and long term, in a real-life setting.
2. Safety of ustekinumab in CD patients.

#### 2.5.2. Secondary Outcomes

1. Effectiveness of intensification of ustekinumab treatment (either by decreasing the intervals of ustekinumab administration or by dose intensification (shortening of the ustekinumab administration interval to less than 8 weeks or administration of a reinduction IV dose of ustekinumab)).
2. Predictive factors of response in a real-life setting.

We evaluated outcomes at weeks 8–14 (short term), 16–24 (medium term), and 48–52 (long term), where available.

### 2.6. Data Synthesis and Statistical Analysis

All analyses were pre-planned a priori. The outcomes were thereafter combined using the inverse variance method, providing 95% confidence intervals (CIs). Due to the expected high heterogeneity in the design and the results of the studies, a random effects model was used. Heterogeneity was analysed using I^2^ statistic: according to I^2^ values, the heterogeneity was considered: not important (I^2^ < 40%), moderate (40–75%), and considerable (>75%). Such interpretations were also adjusted for the magnitude of the effect and/or the strength of the evidence given (i.e., *p*-value <0.1 of the Chi^2^ test). Safety data were reported as the proportion of AEs per patient.

Begg’s funnel plot was used to estimate the possibility of publication bias [13]. Post hoc sensitivity analyses were performed for each meta-analysis subgroup by excluding those studies that were identified as potentially introducing a critical risk of bias that could likely modify the outcome. Data were analysed using the Review Manager software (version 5.4.1, Copenhagen, Denmark).

## 3. Results

### 3.1. Study Selection and Characteristics

**Figure 1:**
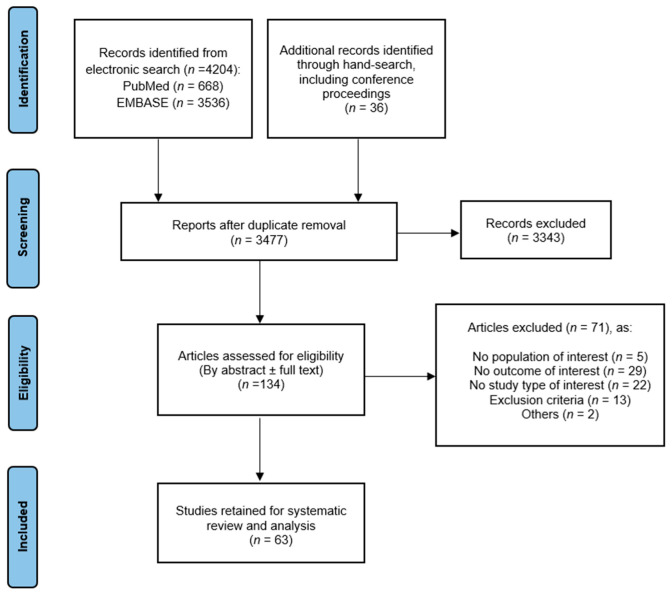
PRISMA flowchart of the screening and selection of relevant studies for inclusion in the meta-analysis.

A total of 63 studies (including 8529 patients) met the inclusion criteria and were finally included in the systematic review and meta-analysis (Figure 1) [14,15,16,17,18,19,20,21,22,23,24,25,26,27,28,29,30,31,32,33,34,35,36,37,38,39,40,41,42,43,44,45,46,47,48,49,50,51,52,53,54,55,56,57,58,59,60,61,62,63,64,65,66,67,68,69,70,71,72,73,74,75,76]. Forty-four (70%) were reported as full-text articles and nineteen (30%) were abstracts.

Of the 63 studies included, 59 (94%) focused on biologic-experienced patients. Only one study focused exclusively on naïve patients [55], and in three studies, prior biologic exposure was not reported. Only three studies (4.8%) included paediatric patients [54,59,63] (Table 1).

### 3.2. Effectiveness of Ustekinumab

The effectiveness of ustekinumab in the selected studies is summarised in Table 2.

#### 3.2.1. Clinical Response

**Figure 2:**
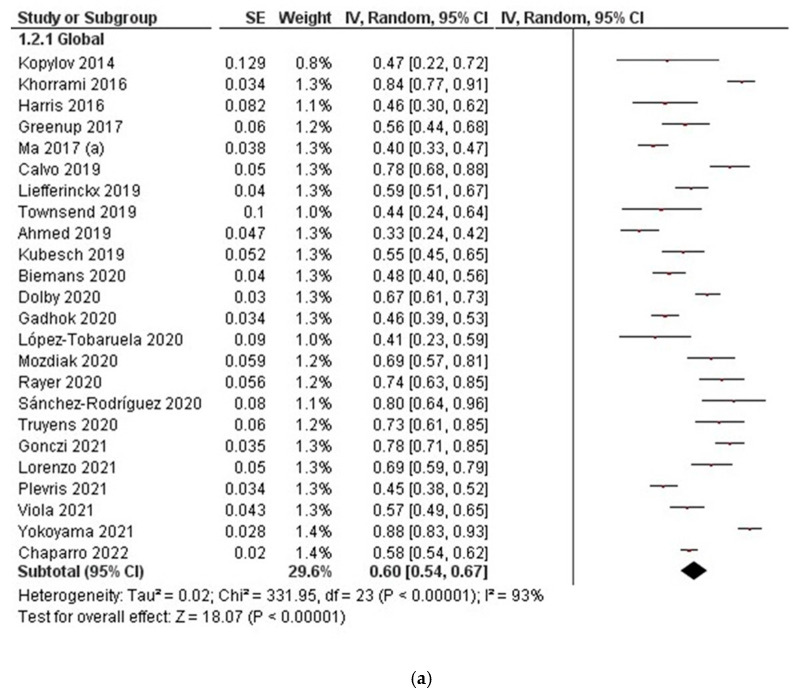

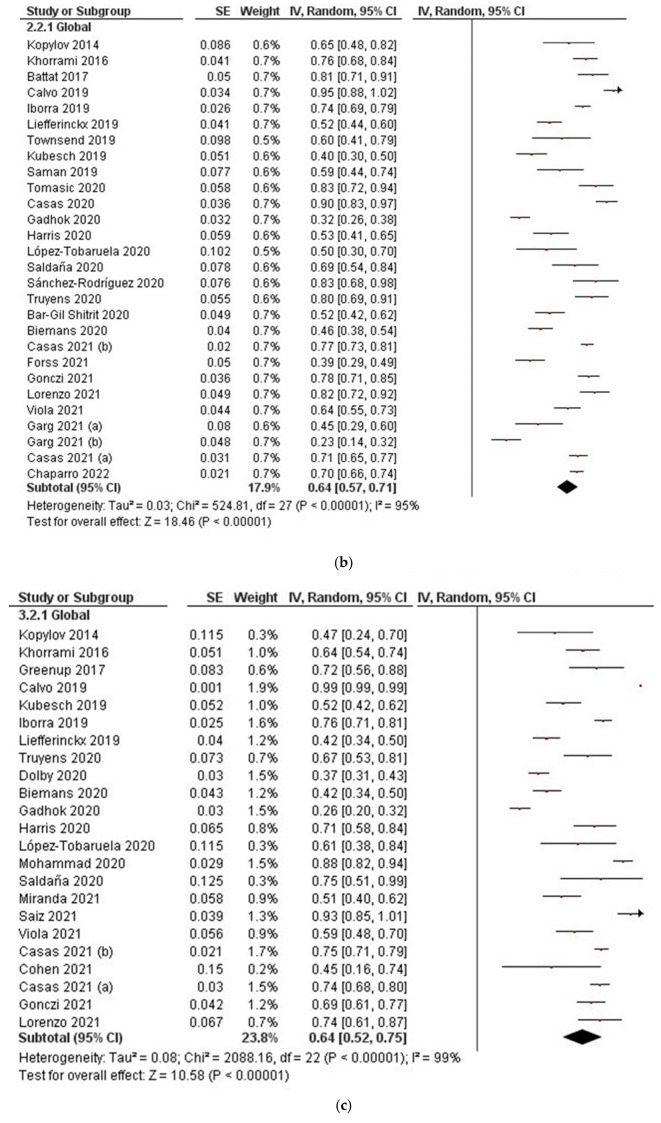
Clinical response to ustekinumab in Crohn’s disease: (**a**) clinical response in the short term (8 w–14 w); (**b**) clinical response in the medium term (16 w–24 w); (**c**) clinical response in long term (48 w–52 w).

The short-term follow-up (8 w–14 w) was analysed for a total of 24 studies. Clinical response rates ranged from 33% to 88%, with an overall pooled rate of 60% (95% CI, 54–67, I^2^ = 93%) (Figure 2a). Combining all 28 studies reporting clinical response data in the medium term (16 w–24 w), clinical response rates ranged from 23% to 95%, with an overall pooled rate of 64% (95% CI, 57–71, I^2^ = 95%) (Figure 2b). Finally, the long-term follow-up (48 w–52 w) was analysed for 23 studies. Clinical response rates ranged from 26% to 99%, with an overall pooled rate of 64% (95% CI, 52–74, I^2^ = 99%) (Figure 2c).

Subgroup analyses for clinical response based on the proportion of patients who were biologic-naïve, by type/design of study (full text vs. abstract; prospective vs. retrospective), by study population (children vs. adults), or by ustekinumab induction regimen did not explain between-study heterogeneity.

#### 3.2.2. Clinical Remission

**Figure 3:**
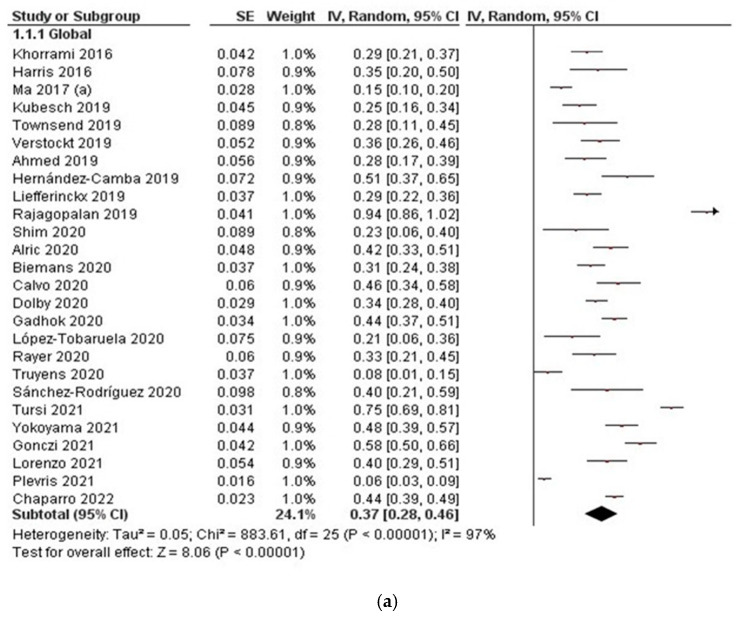

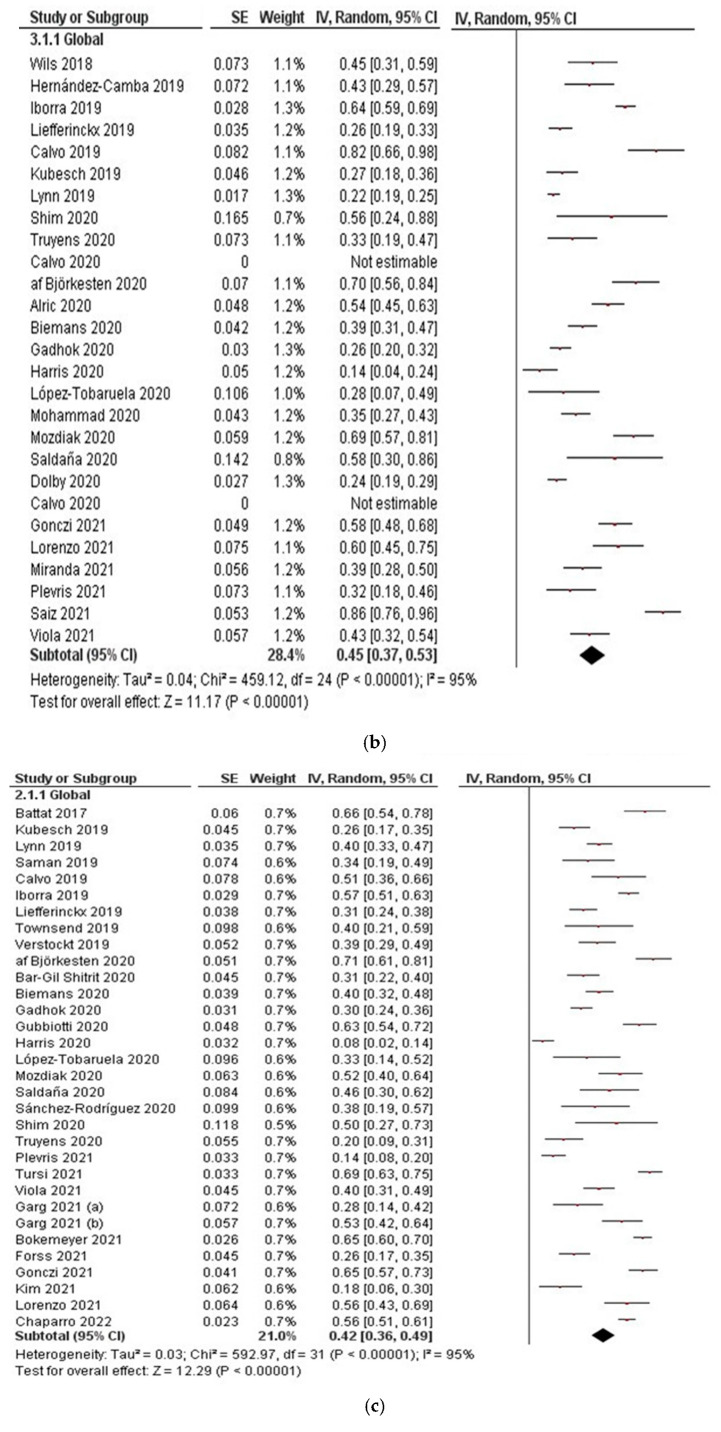
Clinical remission associated with ustekinumab in Crohn’s disease: (**a**) clinical remission in the short term (8 w–14 w); (**b**) clinical remission in the medium term (16 w–24 w); (**c**) clinical remission in the long term (48 w–52 w).

Clinical remission rates of the 26 studies included in the short-term (8 w–14 w) group ranged from 6% to 94%, with an overall pooled rate of 37% (95% CI, 28–46, I^2^ = 97%) (Figure 3a). In the medium term (16 w–24 w), combining 32 studies, the pooled estimate of clinical remission rate was 42% (95% CI, 36–49, I^2^ = 95%). Clinical remission ranged from 8% to 71% (Figure 3b). A total of 25 studies reported clinical remission data in the long term (48 w–52 w). Clinical remission rates ranged from 14% to 86%, with an overall pooled rate of 45% (95% CI, 37–53, I^2^ = 95%) (Figure 3c). All the subgroup analyses for clinical remission showed considerable heterogeneity.

#### 3.2.3. Corticosteroid-Free Clinical Remission

Fifteen studies reported corticosteroid-free clinical remission rates in the short term (8 w–14 w). Corticosteroid-free clinical remission rates ranged from 19% to 56%, with an overall pooled rate of 33% (95% CI, 27–40, I^2^ = 86%) (Figure S2A). In the medium term (16 w–24 w), the overall pooled rate of the 24 studies included was 42% (95% CI, 33–59, I^2^ = 93%), and rates ranged from 7% to 62% (Figure S2B). Finally, combining all 18 studies reporting corticosteroid-free clinical remission data in the long term, the overall pooled rate was 46% (95% CI, 30–59, I^2^ = 98%), with corticosteroid-free clinical remission rates ranging from 15% to 96% (Figure S2C). Subgroup analyses for corticosteroid-free clinical remission did not explain between-study heterogeneity.

#### 3.2.4. Endoscopic Remission

Endoscopic remission was achieved in 24% of the cases (95% CI, 13–34, I^2^ = 96%) (Figure S3A). The remission rates of the 12 studies included in the analysis ranged from 1% to 69%. In the long term, 11 studies reported endoscopic remission data and the overall pooled rate was 33% (95% CI, 25–40, I^2^ = 81%), ranging from 13% to 69% (Figure S3B).

Subgroup analyses for endoscopic remission based on the proportion of patients who were biologic-naïve, by type/design of study (full text vs. abstract/prospective vs. retrospective), by study population (children vs. adult), or by ustekinumab induction regimen did not explain between-study heterogeneity.

### 3.3. Loss of Response to Ustekinumab

The loss of response rates and the median time to loss of response of each study (when available) are detailed in Table 3. The loss of response rate ranged from 0% to 67%, with an overall pooled rate of 18% (95% CI, 14–22, I^2^ = 98%). Ustekinumab secondary loss of response rate in the medium term (six months) was 18% (95% CI, 13–24, I^2^ = 88%). In the long term (12 months), the overall loss of response to ustekinumab rate was 18% (95% CI, 13–23, I^2^ = 98%).

### 3.4. Dose Optimisation

In 32 studies (51%), some type of dose optimisation of ustekinumab was reported (Table 3). The overall need for dose optimisation (dose escalation and/or intensification) was 30% (95% CI, 8–53, I^2^ = 100%), ranging from 4% to 100%. The effectiveness of dose optimisation was 59% (95% IC, 31–86, I^2^ = 100%).

### 3.5. Predictors of Response

Predictors of response were reported in 37 studies (59%). The most frequent predictors of poor clinical response were previous biologic exposure (anti-TNF and/or vedolizumab) [26,39,40,44,47,51,65,72,74], stricturing disease [19,20,39,44,65], penetrating disease [27,39,44,51,65], and high Harvey-Bradshaw index (HBI) at first dose [19,20,51,72,73,75]. Concomitant corticosteroids, extraintestinal manifestations, male sex, old age, smoking, and low body mass index were also reported in some studies. The most frequent predictors of response were concomitant immunomodulators [20,35,67] and ileal [26] or ileocolonic disease [20,28].

### 3.6. Safety

In total, 2191 AEs were reported in 1088 patients, resulting in a pooled estimate of incidence rate of 26% (95% CI, 25–27). Of all AEs, 190 (8.7%) were serious AEs. The most frequent AE was infections [335/8529 (3.9%)], and 27 of the 8529 (0.3%) patients included in the meta-analysis developed malignancy. One hundred and forty-nine AEs (6.8%) led to discontinuation of treatment (Table 4).

### 3.7. Quality of Included Studies and Risk of Publication Bias

Quality of evidence grading of the studies ranged from five (eight studies) to nine (one study). Four studies (9.3%) were graded as ‘high quality’ studies (Newcastle–Ottawa score ≥ 7). The quality of each included study is detailed in Table 5.

Begg’s funnel plot was used, and we did not identify publication bias (Figure S4).

## 4. Discussion

To the best of our knowledge, this systematic review and meta-analysis summarises the largest collection of real-world evidence assessing the effectiveness and safety of ustekinumab in CD, including 63 studies and 8529 patients. Our analysis reports relatively high remission and response rates in refractory CD patients (i.e., those previously exposed to other biological agents), supporting that ustekinumab represents a relevant therapeutic option in the management of CD.

The efficacy and safety of induction therapy with ustekinumab in patients with moderately to severely active CD was demonstrated in the IM-UNITI trials, in which ustekinumab was shown to be effective and safe in the long term (up to 5 years in preliminary data) [6,7,8]. Despite the benefit of ustekinumab in the aforementioned clinical trials, real-world studies are needed to confirm the effectiveness and the safety reported by randomised studies.

Our systematic review almost exclusively includes refractory CD patients: almost all were biologic-experienced, and the vast majority had experienced failure of two or more anti-TNF drugs (Table 1). The overall clinical response and remission rates in our study in the short term were relatively high (60% and 37%, respectively). Moreover, approximately one-third of the patients included in our analysis were in corticosteroid-free clinical remission at week 14. However, the percentage of patients who had a response at week 6 in the UNITI-1 clinical trial was lower (34%) [6]. This difference may be partially explained by the less stringent definition of response in real-world studies included in our review (employing mainly the HBI and/or PGA) in contrast with clinical trials, in which CDAI is generally used. Additionally, short-term clinical response was evaluated after 6 and 8 weeks in the UNITI-1 clinical trial, while in the studies included in our meta-analysis, it was evaluated between 8 and 14 weeks. In the UNITI-1 trial, the one-year remission rate was 41% [6]; in the long term (week 52), 34% of patients in the every-8-weeks group and 29% in the every-12-weeks group were in clinical remission [8]. In the long term, our pooled clinical remission and response rates were 45% and 64% at one year. Additionally, we could not confirm the higher clinical response rates associated with subcutaneous compared with intravenous induction previously reported by Macaluso et al. [77].

Regarding endoscopic outcomes, results should be interpreted with caution, because the number of patients with available endoscopic data was quite limited (Table 2). Currently, data from clinical trials on the ability of ustekinumab to induce mucosal healing are scarce. In IM-UNITI, a sub-study showed rates of endoscopic response and remission of 17% and 11%, respectively [78]. Despite the heterogeneity of our results (due to the different criteria for the assessment of endoscopic response and the limited availability of these data in clinical practice), remarkably significant endoscopic remission rates were reported in the medium (six months, 24%) and in the long term (12 months, 33%).

Previous biologic exposure, stricturing, and penetrating CD and higher HBI at baseline were associated with a lower probability of achieving clinical remission in the observational studies included in our systematic review. While these clinical outcomes are promising (and could be taken into account in the future for the selection of a treatment schedule), controlled studies are necessary to confirm these data.

Medical treatment options for anti-TNF refractory patients are eagerly needed, and ustekinumab has recently emerged as a new therapeutic target. Despite the effectiveness of ustekinumab reported in short- and long-term studies, a significant percentage of patients treated with this drug may lose response (18% in our study). In this scenario, the need for dose optimisation is relatively high in clinical practice, as shown by our results, in which dose optimisation was needed in 30% of cases.

In our review, 60% of the intensified patients achieved clinical remission with different dose optimisation regimens: dose escalation to every 8 weeks and/or intensification every 6–4 weeks, or intravenous reinduction. Reinduction with intravenous ustekinumab after secondary loss of response in CD is a relatively new strategy to regain efficacy. In this scenario, an observational and multicentre study of 53 patients in Spain reported that 49% and 43% of the cohort were in clinical remission at week 8 and week 16 after reinduction, respectively, whereas 64% and 53% had clinical response [79].

Finally, two recently published systematic review and meta-analyses evaluated the safety of ustekinumab in clinical trials [8,80]. Rolston et al. compared rates of AEs in randomised controlled trials of ustekinumab compared to placebo among a spectrum of autoimmune diseases. Of the 30 studies included, 5 were conducted in IBD patients. In this subgroup, there were no differences in the rates of serious or mild/moderate AEs for ustekinumab vs. placebo or in the rates of AEs for low vs. high-dose ustekinumab [80]. Likewise, Sandborn et al. reported that safety events were similar in the placebo and in the ustekinumab groups for all AEs, serious AEs, infections, and serious infections [8].

To our knowledge, the safety of ustekinumab in observational studies was recently summarised in three reviews [77,81,82]. In the systematic review of Macaluso et al., including thirteen studies (1450 patients), the pooled incidence rate of total AEs was 19.1 per 100 patients-year [77]. Similarly, Engel et al. combined the results of 578 patients from six different cohorts, and a total of 134 AEs were reported with a pooled proportion of 21%. Finally, in the study of Honap et al., 498 AEs were reported in 2977 patients (17%) resulting in a pooled estimate of incidence rate of 14% [82].

Our meta-analysis of 8529 patients shows similar results, with a pooled incidence rate of 26%. However, it is necessary to emphasise that AEs were reported heterogeneously in the observational studies included. We defined serious AEs following the criteria of the FDA to minimise this bias. Thus, despite the heterogeneity of reported AEs, our study confirms that the proportion of serious AEs was low, and only a minority of them (6.8%) led to discontinuation of ustekinumab treatment.

The limitations of the present study are mainly those associated with observational studies. Firstly, the majority of included studies had a retrospective study design and, as such, were susceptible to bias and confounding. Secondly, it is necessary to underline the high degree of between-study statistical heterogeneity in data analyses. In our systematic review and meta-analysis, data were stratified by study design, population included, administered dose of ustekinumab, and prior treatment with biological drugs. All the performed sub-analyses did not adequately explain between-study heterogeneity.

Nevertheless, our study has several strengths. First, this is the largest and longest real-world meta-analysis evaluating the effectiveness and safety of ustekinumab in CD published to date. Furthermore, we also provide data regarding the need for dose optimisation and its effectiveness, which is also a relevant issue in clinical practice.

In conclusion, our meta-analysis emphasises that ustekinumab is effective and safe for the treatment of refractory CD, and that its clinical benefit seems to be higher in real-life observational studies compared with controlled clinical trials. Finally, dose optimisation of ustekinumab is frequently required in clinical practice, and achieves clinical response in a high percentage of cases.

## Figures and Tables

**Table 1:** Baseline characteristics of the studies included in the meta-analysis.

Authors	Year	Abstract or Full Text	N	Design	Period	Median Follow-Up	Adult or Children	Dose UST (Induction ± Maintenance)	Prior IMM	Prior Biologic			Concomitant CE	Concomitant IMM
										≥1 Biologic	Anti-TNF Failure	Anti-TNF + Vedolizumab Failure		
Kopylov [14]	2014	F	38	R	March 2011 to November 2013	32 w	Ad	sc + 90 mg sc q8w	38/38 (100%)	38/38 (100%)	1 anti-TNF: 38/38 (100%) ≥2 anti-TNF: 36/38 (95%)	NR	22/38 (57.8%)	4/38 (10.6%)
Harris [15]	2016	F	45	R	June 2011 to June 2014	12 w	Ad	sc + 90 mg sc q8w	NR	45/45 (100%)	1 anti-TNF: 45/45 (100%) ≥2 anti-TNF: 44/45 (98%)	NR	32/45 (71%)	29/45 (65%)
Khorrami [16]	2016	F	116	R	March 2011 to December 2014	40 w	Ad	sc + 90 mg sc q8w	116/116 (100%)	116/116 (100%)	1 anti-TNF 116/116 (100%) ≥2 anti-TNF: 101/116 (87.1%)	NR	37/116 (31.9%)	42/116 (36.2%)
Battat [17]	2017	F	62	P	April 2014 to September 2015	NR	Ad	sc+ 90 mg sc q8w	NR	61/62 (98.4%)	1 anti-TNF: 61/62 (98.4%)	NR	19/62 (30.7%)	Thiopurines: 10/62 (16.1%) Methotrexate: 6/62 (9.7%)
Greenup [18]	2017	F	73	R	May 2013 to November 2016	NR	Ad	sc + 90 mg q8w	NR	72/73 (99%)	1 anti-TNF 72/73 (99%) ≥2 anti-TNF 9/73 (12.5%)	NR	19/73 (26%)	30/73 (42%)
Ma (a) [19]	2017	F	167	R	January 2011 to July 2016	45 w	Ad	sc/IV + 90 mg sc q8w	126/167 (75.4%)	159/167 (95.2%)	1 anti-TNF: 117/167 (70.1%) ≥2 anti-TNF: 93/167 (55.7%)	8/167 (4.8%)	72/167 (43.1%)	73/167 (43.7%)
Ma (b) [20]	2017	F	104	R	January 2011 to July 2016	57.2 w	Ad	90 mg sc q8w-q12w-q6w	NR	96/104 (92.3%)	1 anti-TNF: 96/104 (92.3%)	NR	40/104 (38.5%)	44/104 (42.3%)
Wils [21]	2018	F	88	R	March 2011 to December 2014	106.4 w	Ad	90 mg sc q8w-q12w	86/88 (98%)	88/88 (100%)	1 anti-TNF: 88/88 (100%) ≥2 anti-TNF: 79/88 (90%)	0/88 (0%)	13/88 (15%)	26/88 (30%)
Ahmed [22]	2019	F	66	R/P	2014 to 2017	16 w	Ad	NR	Thiopurines: 23/66 (34.8%) Methotrexate: 7/66 (10.6%)	56/66 (84.8%)	1 anti-TNF: 56/66 (84.8%)	19/66 (28.8%)	NR	NR
Calvo [23]	2019	A	68	R	April 2010 to April 2019	76 w	Ad	IV + 90 mg sc q8w-q12w	61/68 (90%)	68/68 (100%)	1 anti-TNF: 67/68 (98%) ≥2 anti-TNF: 47/68 (69%)	11/68 (16%)	12/68 (18%)	15/68 (22%)
Hernández Camba [24]	2019	A	47	R	June 2017 to June 2018	NR	Ad	IV + 90 mg sc q12w	NR	39/47 (82.6%)	NR	NR	NR	43/47 (91%)
Hoffmann [25]	2019	F	57	R	December 2016 to March 2018	32 w	Ad	IV + 90 mg sc q8w-q12w	47/57 (82.5%)	54/57 (94.7%)	NR	NR	20/57 (35.1%)	3/57 (5.3%)
Iborra [26]	2019	F	407	R	Since June 2017	NR	Ad	IV + 90 mg sc q4w-q8w-q12w	NR	389/407 (96%)	1 anti-TNF: 389/407 (96%) ≥2 anti-TNF: 248/407 (61%)	88/407 (22%)	135/407 (33.2%)	147/407 (36.1%)
Kubesch [27]	2019	F	106	R	NR	49.1 w	Ad	IV + 90 mg sc q8-q12w	95/106 (89.6%)	102/106 (96.2%)	1 anti-TNF: 55/106 (51.9%) ≥2 anti-TNF: 46/106 (43.4%)	36/106 (34.4%)	38/106 (35.8%)	NR
Liefferinckx [28]	2019	F	152	R	September 2016 to August 2017	NR	Ad	IV + 90 mg sc q8w	NR	151/152 (99.3%)	1 anti-TNF: 151/152 (99.3%) ≥2 anti-TNF: 124/152 (82%)	106/152 (69.7%)	68/152 (44.7%)	25/152 (16.4%)
Lynn [29]	2019	A	594	R	NR	NR	Ad	NR	NR	559/594 (94%)	1 anti-TNF: 309/594 (52%)	238/594 (40%)	NR	NR
Rajagopalan [30]	2019	A	33	R	May 2017 to January 2019	12 w (mean)	Ad	NR	NR	31/33 (94%)	NR	NR	NR	NR
Saman [31]	2019	F	41	R	December 2016 to July 2018	32 w	Ad	IV + 90 mg sc q8w-q12w	38/41 (92.7%)	38/41 (92.7%)	1 anti-TNF: 28/41 (68.3%) ≥2 anti-TNF: 10/41 (24.4%)	10/41 (24.4%)	15/41 (36.6%)	NR
Townsend [32]	2020	F	45	R	NR	NR	Ad	IV + 90 mg sc q8w	NR	45/45 (100%)	1 anti-TNF: 45/45 (100%) ≥2 anti-TNF: 12 (26.7%)	NR	19/45 (42.2)	16 (35.6)
Verstockt [33]	2019	F	86	P	September 2016 to January 2018	32 w	Ad	IV + 90 mg sc q8w	NR	82/86 (95.3%)	1 anti-TNF: 82 (95.3%)	58/86 (67.4%)	30/86 (34.9%)	2/86 (2.4%)
af Björkesten [34]	2020	F	155	R	January 2017 to December 2018	56.8 w	Ad	IV + 90 mg sc q8w-q12w	57/155 (36.8%)	150/155 (96.8%)	NR	61/155 (39.4%)	NR	NR
Alric [35]	2020	F	107	R	December 2016 to August 2018	NR	Ad	IV + 90 mg sc q8w-q12w	Thiopurines: 94/107 (87.9%) Methotrexate: 35/107 (32.7%)	83/107 (77.6%)	1 anti-TNF: 83/107 (77.6%) ≥2 anti-TNF: 58/107 (54.2%)	NR	30/107 (28%)	21/107 (19.6%)
Bar-Gil Shitrit [36]	2020	F	106	P	NR	NR	Ad	IV + 90 mg sc q8w	45/106 (41.7%)	106/106 (100%)	NR	NR	NR	29/106 (26.9%)
Bennett [37]	2020	F	96	R	September 2009 to November 2017	40.3 w (IV reinduction) 62.9w (sc reinduction)	Ad	sc/IV + 90 mg sc q8w	NR	96/96 (100%)	1 anti-TNF: 96/96 (100%) ≥2 anti-TNF: 58/96 (60%)	31/96 (32%)	33/96 (34.4%)	43/96 (44.8%)
Biemans [38]	2020	F	221	P	December 2016 to January 2019	48 w	Ad *	IV + 90 mg sc q8w-q12w	216/221 (97.7%)	218/221 (98.6%)	1 anti-TNF: 218/221 (98.6%) ≥2 anti-TNF: 162/221 (73.3%)	102/221 [46.2]	35/221 (15.8%)	44/212 (19.9%)
Calvo [39]	2020	A	28	R	April 2017 to April 2019	76 w	Ad	IV + 90 mg sc q8w-q12w	NR	28/28 (100%)	NR	NR	NR	NR
Casas [40]	2020	F	69	R	NR	32 w	Ad	IV/sc + 90 mg sc q8w	NR	69/69 (100%)	NR	12/69 (17%)	20/69 (29%)	15/69 (22%)
Gadhok [41]	2020	A	211	NR	October 2016 to October 2018	NR	Ad	IV + 90 mg sc q8w-q12w	NR	207/211 (96%)	NR	NR	NR	49/211 (23%)
Gubbiotti [42]	2020	A	104	NR	NR	32 w	Ad	IV + 90 mg sc q8w-q12w	NR	104/104 (100%)	NR	NR	31/104 (29.7%)	NR
Harris [43]	2020	F	84	R	Up to December 2018	27,809 (treatment days)	Ad	IV + 90 mg sc q8w-q12w	NR	82/84 (97.6%)	1 anti-TNF: 81/84 (96.4%)	35/84 (42%)	6/84 (7.1%)	38/84 (45.2%)
Kakkadasam [44]	2020	A	76	R	June 2017 to July 2019	61 w	Ad	IV + 90 mg sc q8w	NR	50/76 (65.8%)	1 anti-TNF: 49/76 (64.5%)	10/76 (13.1%)	38/76 (50%)	32/76 (42.1%)
Kopylov [45]	2020	F	142	R	NR	26 w	Ad	IV + 90 mg sc q8w	NR	137/142 (96.5%)	NR	57/142 (40%)	34/142 (24%)	24/142 (16.9%)
López- Tobaruela [46]	2020	A	37	R	NR	50 w (mean)	Ad	NR	35/37 (94.6%)	35/37 (94.6%)	1 anti-TNF: 35/37 (94.6%) ≥2 anti-TNF: 23/37 (65.7%)	4/37 (10.8%)	11/37 (29.7%)	12/37 (32.4%)
Mohammad [47]	2020	A	123	R	January 2017 to August 2019	NR	Ad	NR	NR	98/123 (79.5%)	1 anti-TNF: 98/123 (79.5%)	21/123 (17.1%)	NR	NR
Monin [48]	2020	F	156	R	October 2016 to May 2020	60 w	Ad	IV + 90 mg sc q8w	111/148 (75.5%)	113/148 (76.4%)	NR	110/148 (74.3%)	51/148 (34.5%)	20/148 (13.5%)
Mozdiak [49]	2020	A	62	R	NR	NR	Ad	IV + 90 mg sc q8w	NR	60/62 (97%)	NR	NR	NR	19/62 (30.6%)
Rayer [50]	2020	A	61	R	NR	67 w (mean)	Ad	NR	NR	NR	NR	NR	NR	NR
Parra [51]	2022	F	245	R	November 2017 to November 2019	up to 56 w	Ad	IV + 90 mg sc q8w	50/204 (25%)	212/245 (86.5%)	1 anti-TNF:182/245 (74.3%)	NR	135/245 (60.5%)	54/245 (22.1%)
Saldaña [52]	2020	F	61	P	August 2017 to February 2019	NR	Ad	IV + 90 mg sc q8w	NR	61/61 (100%)	NR	9/61 (48%)	10/61 (16.4%)	16/61 (26.2%)
Sánchez-Rodríguez [53]	2020	A	25	R	June 2017 to May 2019	53.3 w	Ad	IV + 90 mg sc q8w	25/25 (100%)	24/25 (96%)	NR	NR	NR	NR
Shim [54]	2020	A	22	NR	NR	NR	Both	NR	NR	19/22 (86.4%)	NR	NR	6/22 (27.3%)	13/22 (59.1%)
Tomasic [55]	2020	A	42	R	January 2018 to April 2020	64 w	Ad	NR	NR	0/42 (0%)	0/42 (0%)	0/42 (0%)	NR	NR
Truyens [56]	2020	A	67	R	December 2017 to August 2019	60 w	Ad	IV + 90 mg sc q8w	NR	62/67 (92.5%)	NR	NR	29/67 (43.3%)	14/67 (20.9%)
Bokemeyer [57]	2021	A	339	P	January 2017 to December 2020	NR	NR	NR	NR	305/339 (90%)	NR	NR	NR	NR
Casas [58]	2021	A	648 >60 y: 212 <60 y: 436	R	NR	NR	NR	NR	NR	>65y: 180/212 (84.8%) <65y: 421/436 (96.7%)	NR	NR	>65y: 54/212 (25.5%) <65y: 127/436 (29.3%)	NR
Cohen [59]	2021	F	11	R	December 2015 to July 2018	24–88 w	C	sc/IV + 90 mg sc q8w	10/11 (90.9%)	11/11 (100%)	1 anti-TNF: 11/11 (100%) ≥2 anti-TNF: 3/11 (27.3%)	NR	4/11 (36.4%)	4/11 (36.4%)
Forss [60]	2021	F	114	P	January 2017 to November 2018	NR	Ad	IV + 90 mg sc q8w-q12w	NR	107/112 (94%)	NR	NR	21/114 (18%)	26/114 (23%)
Garg [61]	2021	F	117 >65 y: 39 <65 y: 78	R	September 2016 to September 2019	>65 y: 70 w (mean) <65 y: 70 w (mean)	Ad	IV + 90 mg sc q12w	>65y: 19/39 (48.7%) <65: 41/78 (52.6%)	>65y: 37/39 (94.9%) <65y: 77/78 (98.7%)	NR	>65y: 0/39 (0%) <65y: 7/78 (8.6%)	>65y: 20/39 (51.3%) <65y: 37/78 (47.4%)	>65y: 2/39 (5.1%) <65y: 11/78 (14.1%)
Gonczi [62]	2021	F	142	P	January 2019 to May 2020	60 w	Ad	IV + 90 mg sc q12w	115/142 (80.9%)	138/142 (97.2%)	1 anti-TNF: 138/142 (97.2%) ≥2 anti-TNF: 90/142 (63.1%)	36/142 (25.5%)	48/142 (34%)	29/142 (20.2%)
Kim [63]	2021	F	38	R	January 2016 to December 2019	62.1 w	C *	IV + 90 mg sc q8w	17/38 (44.7%)	38/38 (100%)	1 anti-TNF: 38/38 (100%) ≥2 anti-TNF: 13/38 (34.2%)	5/38 (13.2%)	7/38 (18.4%)	NR
Lorenzo [64]	2021	F	98	R	July 2017 to December 2019	28 w (mean)	Ad	IV + 90 mg sc q8w	Thiopurines: 81/98 (91%) Methotrexate: 48/98 (49%)	97/98 (99%)	NR	NR	27/98 (27.5%)	Thiopurines: 13/98 (13.9%) Methotrexate: 2/98 (2.0%)
Manlay [65]	2021	F	224	R	July 2014 to May 2020	66 w (mean)	Ad	IV + 90 mg sc q8w	Thiopurines: 159/224 (70.9%) Methotrexate: 39/224 (17.4%)	224/224 (100%)	NR	54/224 (24.1%)	59/224 (26.3%)	32/224 (14.3%)
Miranda [66]	2021	F	92	P	NR	NR	Ad	IV + 90 mg sc q8w	NR	85/92 (92.4%)	NR	6/92 (6.5%)	NR	NR
Plevris [67]	2021	F	216	R	July 2017 to December 2019	35 w	Ad	IV + 90 mg sc q8w	NR	213/216 (98.6%)	NR	NR	88/216 (40.7%)	55/216 (25.5%)
Saiz [68]	2021	A	49	R	January 2013 to March 2020	112 w	Ad	IV/sc + 90 mg sc q8w	NR	49/49 (100%)	1 anti-TNF: 49/49 (100%) ≥2 anti-TNF: 13/49 (27%)	NR	NR	35/49 (71.4%)
Scribano [69]	2021	F	140	R	November 2018 to February 2020	NR	Ad	IV + 90 mg sc q8w-q12w	NR	140/140 (100%)	1 anti-TNF: 140/140 (100%) ≥2 anti-TNF 38/140 (27.1%)	28/140 (20%)	22/140 (15.7%)	12/140 (8.6%)
Sipponen [70]	2021	F	155	R	January 2017 to December 2018	62.8 w (mean, intensification) 50.8 w (mean, no intensif.)	Ad	IV + 90 mg sc q8w-q12w	NR	150/155 (96.8%)	NR	NR	64/155 (41.3%)	51/155 (32.9%)
Straatmijer [71]	2021	F	252	P	NR	NR	Ad	IV + 90 mg sc q8w-q12w	NR	50/252 (99.2%)	1 anti-TNF: 250/252 (99.2%) ≥2 anti-TNF: 184/252 (73%)	108/252 (42.9)	NR	NR
Tursi [72]	2021	F	194	R	Until December 2019	24 w (mean)	Ad	IV + 90 mg sc q8w	121/194 (62.4%)	147/194 (75.8%)	NR	47/194 (24.2%)	177/194 (91.2%)	NR
Viola [73]	2021	F	131	P	January 2019 to August 2019	NR	Ad	IV + 90 mg sc q8w-q12w	NR	130/131 (99%)	≥2 anti-TNF: 38/131 (29%)	46/131 (35%)	56/131 (43%)	14/131 (11%)
Yokoyama [74]	2021	F	341	P	May 2017 to June 2020	NR	Ad *	IV + 90 mg sc q8w-q12w	72/339 (24.8%)	245/341 (72.3%)	NR	1/341 (0.4%)	104/339 (30.7%)	68/339 (20.1%)
Chaparro [75]	2022	F	463	R	Before July 2018	62 w	Ad	IV + 90 mg sc q8-q12w	162/463 (35%)	447/463 (96.5%)	1 anti-TNF: 374/463 (83.7%)	109/463 (24.4%)	NR	162/463 (35%)
Lenti [76]	2022	F	259	R	NR	NR	Ad	NR	NR	209/259 (80.7%)	NR	78/259 (30.1%)	NR	NR

* Adult and children. F: full text; A: abstract; P: prospective; R: retrospective; Ad: adult; C: children; IV: intravenous; sc: subcutaneous; NR: not reported; CE: corticosteroids; anti-TNF: anti-tumour necrosis factor; IMM: immunomodulators; UST: ustekinumab.

**Table 2:** Effectiveness of ustekinumab.

Authors	Clinical Response				Clinical Remission				CE-Free Clinical Remission				Endoscopic Remission	
	w8–w14	w24–w36	w48–w52	w52–w104	w8–w14	w24–w36	w48–w52	w52–w104	w8–w14	w24–w36	w48–w52	w52–w104	w24–w36	w48–w52
Kopylov [14]	28/38 (73.7%)	20/31 (64.5%)	9/19 (47.4%)	NR	NR	NR	NR	NR	NR	NR	NR	NR	2/13 (15.4%)	NR
Harris [15]	17/37 (46%)	NR	NR	NR	13/37 (35%)	NR	NR	NR	NR	NR	NR	NR	NR	NR
Khorrami [16]	97/116 (73.6%)	81/106 (76.4%)	56/88 (63.6%)	NR	33/116 (28.4%)	NR	NR	NR	NR	NR	NR	NR	NR	NR
Battat [17]	NR	50/62 (80.7%)	NR	NR	NR	41/62 (66.1%)	NR	NR	NR	31/62 (50%)	NR	NR	11/56 (19.6%)	NR
Greenup [18]	38/68 (56%)	NR	21/29 (72%)	NR	NR	NR	NR	NR	9/19 (47%)	NR	NR	NR	NR	NR
Ma (a) [19]	65/167 (38.9%)	NR	NR	NR	25/167 (15%)	NR	NR	NR	NR	NR	31/111 (27.9%)	NR	NR	25/92 (27.2%)
Ma (b) [20]	NR	NR	NR	NR	NR	NR	NR	NR	NR	NR	NR	NR	NR	36/94 (38.3%)
Wils [21]	NR	NR	NR	47/47 (100%)	NR	NR	21/47 (45%)	NR	NR	NR	NR	NR	NR	NR
Ahmed [22]	33/66 (50%)	NR	NR	NR	18/65 (27.2%)	NR	NR	NR	NR	NR	NR	NR	NR	NR
Calvo [23]	53/68 (78%)	39/41 (95%)	22/22 (100%)	NR	31/68 (45%)	21/41 (71%)	18/22 (82%)	NR	NR	NR	NR	NR	NR	NR
Hernández-Camba [24]	NR	NR	NR	NR	24/47 (51.6%)	NR	20/47 (42%)	NR	38/47 (80%)	NR	NR	NR	NR	NR
Hoffmann [25]	NR	NR	NR	NR	NR	NR	NR	NR	NR	20/57 (35.1%)	NR	NR	0/6 (0%)	7/17 (41.1%)
Iborra [26]	NR	218/295 (73.9%)	225/295 (76.3%)	NR	NR	169/295 (57.3%)	190/295 (64.4%)	NR	NR	NR	80/135 (59%)	NR	NR	25/159 (15.7%)
Kubesch [27]	51/93 (54.8%)	37/93 (39.8%)	48/93 (51.6%)	23/93 (24.7%)	24/93 (24.7%)	19/93 (20.4%)	25/93 (26.9%)	16/93 (17.2%)	18/93 (19.3%)	NR	19/93 (20.4%)	NR	NR	NR
Liefferinckx [28]	90/152 (59.2%)	79/152 (51.9%)	64/152 (42.1%)	NR	44/152 (28.2%)	47/152 (30.9%)	39/152 (25.7%)	NR	30/152 (19.7%)	41/152 (26.9%)	37/152 (24.3%)	NR	NR	NR
Lynn [29]	NR	NR	NR	NR	NR	77/594 (13%)	130/594 (22%)	NR	NR	NR	NR	NR	101/594 (17%)	202/594 (34%)
Rajagopalan [30]	NR	NR	NR	NR	31/33 (96%)	NR	NR	NR	NR	NR	NR	NR	NR	NR
Saman [31]	NR	24/41 (58.3%)	NR	NR	NR	14/41 (34.1%)	NR	NR	NR	NR	NR	NR	NR	NR
Townsend [32]	22/45 (48.9%)	22/45 (48.9%)	24/45 (53.3%)	NR	16/45 (35.6%)	18/45 (40%)	19/45 (42.2%)	NR	13/45 (28.9%)	17/45 (37.8%)	19/45 (42.2%)	NR	NR	NR
Verstockt [33]	NR	NR	NR	NR	31/86 (36%)	34/86 (39.5%)	NR	NR	27/86 (31.4%)	33/86 (38.4%)	NR	NR	6/86 (7.1%)	NR
af Björkesten [34]	NR	NR	NR	NR	NR	55/78 (70.5%)	30/43 (69.8%)	NR	NR	NR	NR	NR	6/17 (35.3%)	6/18 (33.3%)
Alric [35]	NR	NR	NR	NR	45/107 (42.3%)	NR	58/107 (54.4%)	NR	41/107 (38.2%)	NR	48/107 (44.7%)	NR	NR	NR
Bar-Gil Shitrit [36]	NR	55/106 (52%)	NR	NR	NR	3/106 (31.1%)	NR	NR	NR	4/37 (10.8%)	NR	NR	NR	NR
Bennett [37]	NR	NR	NR	NR	NR	NR	NR	NR	NR	NR	NR	NR	13/51 (25%)	NR
Biemans [38]	73/153 (47.7%)	70/152 (46.1%)	56/132 (42.4%)	NR	47/153 (30.7%)	61/152 (40.1%)	52/132 (39.4%)	NR	37/153 (24.2%)	58/152 (38.2%)	49/132 (37.1%)	NR	NR	NR
Calvo [39]	NR	NR	NR	NR	NR	NR	NR	NR	NR	NR	NR	NR	NR	18/28 (18%)
Casas [40]	NR	62/69 (89.9%)	NR	NR	NR	NR	NR	NR	NR	5/69 (7%)	NR	NR	NR	NR
Gadhok [41]	97/211 (46.2%)	67/211 (31.9%)	53/211 (25.3%)	NR	92/211 (43.5%)	63/211 (29.7%)	54/211 (25.7%)	NR	85/211 (40.3%)	59/211 (28.1%)	54/211 (25.7%)	NR	NR	NR
Gubbiotti [42]	NR	NR	NR	NR	NR	65/104 (62.3%)	NR	NR	NR	53/104 (50.7%)	NR	NR	NR	NR
Harris [43]	NR	38/72 (53%)	35/49 (71%)	NR	NR	6/72 (8%)	7/49 (14%)	NR	NR	NR	31/49 (65%)	NR	NR	NR
Kakkadasam [44]	NR	NR	NR	NR	NR	NR	NR	NR	NR	35/76 (46%)	NR	NR	NR	NR
Kopylov [45]	NR	NR	NR	NR	NR	NR	NR	NR	NR	NR	NR	NR	NR	NR
López-Tobaruela [46]	12/29 (41.4%)	12/24 (50%)	11/18 (61.1%)	NR	6/29 (20.7%)	8/24 (33.3%)	5/18 (27.8%)	NR	NR	NR	NR	NR	NR	NR
Mohammad [47]	NR	NR	108/123 (88%)	NR	NR	NR	43/123 (35%)	NR	NR	NR	NR	NR	NR	NR
Monin [48]	NR	NR	NR	NR	NR	NR	NR	NR	NR	NR	NR	NR	NR	NR
Mozdiak [49]	43/62 (69%)	NR	NR	NR	NR	32/62 (52%)	43/62 (69%)	NR	NR	NR	NR	NR	NR	NR
Rayer [50]	45/61 (74%)	NR	NR	NR	20/61 (33%)	NR	NR	NR	NR	NR	NR	NR	NR	NR
Parra [51]	189/239 (79.1%)	NR	NR	NR	98/239 (41%)	165/239 (68.9%)	209/235 (87.3%)	NR	NR	NR	80/135 (59.3%)	NR	NR	NR
Saldaña [52]	NR	24/35 (69.9%)	9/12 (75%)	NR	NR	16/35 (45.7%)	7/12 (58.3%)	NR	NR	16/35 (45.7%)	7/12 (58.3%)	NR	NR	NR
Sánchez-Rodríguez [53]	20/25 (80%)	20/24 (83.3%)	NR	NR	10/25 (40%)	9/24 (37.5%)	NR	NR	6/25 (24%)	7/24 (29.2%)	16/18 (88.9%)	NR	NR	NR
Shim [54]	NR	NR	NR	NR	5/22 (22.7%)	9/18 (50%)	5/9 (55.6%)	NR	NR	NR	NR	NR	NR	2/5 (40%)
Tomasic [55]	NR	35/42 (84%)	NR	30/42 (71.4%)	NR	NR	NR	NR	NR	16/42 (38.1%)	NR	15/42 (35.7%)	NR	NR
Truyens [56]	38/52 (73.1%)	43/54 (79.6%)	28/42 (66.7%)	NR	4/52 (7.7%)	11/54 (20.4%)	14/42 (42.4%)	NR	NR	NR	NR	NR	1/16 (6.3%)	2/16 (12.5%)
Bokemeyer [57]	NR	NR	NR	NR	NR	222/339 (65.5%)	NR	NR	NR	181/339 (53.4%)	NR	NR	NR	NR
Casas (a) [58]	NR	150/212 (70.5%)	157/212 (74%)	NR	NR	NR	-	NR	NR	NR	NR	NR	NR	NR
Casas (b) [58]	NR	334/436 (76.6%)	327/436 (74.9%)	NR	NR	NR	NR	NR	NR	NR	NR	NR	NR	NR
Cohen [59]	NR	NR	5/11 (45.5%)	NR	NR	NR	NR	NR	NR	NR	NR	NR	NR	NR
Forss [60]	NR	38/96 (40%)	NR	NR	NR	25/96 (26%)	NR	NR	NR	NR	NR	NR	NR	NR
Garg (a) [61]	NR	18/39 (46.2%)	NR	NR	NR	11/39 (28.2%)	NR	NR	NR	6/20 (30%)	NR	NR	18/71 (25.9%)	NR
Garg (b) [61]	NR	18/78 (23.1%)	NR	NR	NR	41/78 (52.6%)	NR	NR	NR	20/37 (54.1%)	NR	NR	21/71 (29.5%)	NR
Gonczi [62]	107/137 (78.1%)	106/136 (77.9%)	84/122 (69%)	NR	79/137 (57.7%)	88/136 (64.7%)	58/100 (58%)	NR	56/128 (43.8%)	76/132 (57.6%)	47/92 (51.1%)	NR	NR	NR
Kim [63]	NR	NR	NR	NR	NR	7/38 (18.4%)	NR	23/38 (60.5%)	NR	NR	NR	NR	NR	NR
Lorenzo [64]	58/84 (69%)	50/61 (82%)	32/43 (73.7%)	NR	34/84 (40.8%)	34/61 (56%)	26/43 (60.5%)	NR	27/84 (32.4%)	27/61 (44%)	20/43 (47.4%)	NR	NR	NR
Manlay [65]	NR	NR	NR	NR	NR	NR	NR	NR	111/198 (56.1%)	100/161 (62.1%)	104/206 (50.6%)	NR	NR	NR
Miranda [66]	NR	NR	38/75 (50,5%)	NR	NR	NR	29/75 (39%)	NR	NR	NR	NR	NR	NR	26/75 (34%)
Plevris [67]	98/216 (45.4%)	NR	NR	NR	13/216 (6%)	15/108 (13.5%)	13/41 (32%)	NR	NR	NR	NR	NR	7/67 (10.8%)	6/19 (32.7%)
Saiz [68]	NR	NR	40/43 (93%)	13/21 (62%) *	NR	NR	37/43 (86%)	11/21 (52%) *	NR	NR	NR	NR	NR	NR
Scribano [69]	NR	NR	NR	NR	NR	NR	NR	NR	NR	85/140 (61%)	46/140 (64.2%)	NR	NR	NR
Sipponen [70]	NR	NR	NR	NR	NR	NR	NR	NR	NR	NR	NR	NR	NR	NR
Straatmijer [71]	NR	NR	NR	NR	NR	NR	NR	NR	81/251 (32.3%)	104/251 (41.4%)	97/249 (39%)	84/247 (34%)	NR	NR
Tursi [72]	NR	NR	NR	NR	146/194 (75.2%)	135/194 (69.9%)	NR	NR	NR	115/191 (59.3%)	NR	NR	33/62 (53.2%)	NR
Viola [73]	75/131 (68%)	75/117 (64%)	45/76 (59%)	NR	NR	47/117 (40%)	33/76 (43%)	NR	46/131 (35%)	NR	73/76 (96%)	NR	NR	NR
Yokoyama [74]	115/130 (88.5%)	NR	NR	NR	63/130 (48.5%)	NR	NR	NR	NR	NR	NR	NR	NR	NR
Chaparro [75]	268/463 (58%)	320/457 (70%)	NR	NR	204/463 (44%)	256/457 (56%)	NR	113/272 (41.5%)	NR	247/463 (53.3%)	222/437 (50.8%)	97/272 (35.7%)	NR	NR
Lenti [76]	173/259 (66.8%)	NR	97/259 (37.5%)	NR	89/259 (34.4%)	NR	63/259 (24.3%)	NR	NR	NR	NR	NR	NR	NR

* CE: corticosteroids; NR: not reported.

**Table 3:** Loss of response to ustekinumab and frequency and effectiveness of ustekinumab dose optimisation.

Author	Loss of Response Rate (n/N, %)	Median Time to Loss of Response	Dose Optimisation (Dose Escalation and/or Intensification) **	Time to Dose Optimisation (Median)	Effectiveness of Dose Optimisation
Kopylov [14]	NR	NR	Dose escalation: 18/38 (47.4%)	NR	11/18 (61.1%)
Harris [15]	2/45 (4.4%)	NR	NR	NR	NR
Khorrami [16]	29/116 (25%)	NR	Intensification: 11/116 (9.5%)	NR	8/11 (73%)
Greenup [18]	12/42 (8.6%)	88 w	Intensification: 16/62 (25.8%)	NR	3/16 (19%)
Ma (a) [19]	15/167 (9%)	29.3 w	NR	NR	NR
Ma (b) [20]	35/104 (33.7%)	47.4 w	Dose optimisation: 24/104 (23%) * Dose escalation: 17/104 (16.3%) * IV reinduction + dose escalation: 7/104 (6.7%)	Dose escalation: 49.6 w IV reinduction + dose escalation: 84.3 w	Clinical response: * Dose escalation: 9/17 (52.9%) * IV reinduction + dose escalation: 4/7 (57.1%)
Wils [21]	27/88 (30.7%)	NR	32/88 (36.4%)	106.4 w	18/36 (56%)
Calvo [23]	6/68 (8.8%)	20 w	Intensification: 8/68 (15%)	52 w	Clinical response: 4/8 (50%) Clinical remission: 1/8 (13%)
Hernández-Camba [24]	14/47 (30%)	26.8 w (mean)	Dose escalation: 38/47 (80%)	NR	NR
Hoffmann [25]	5/48 (10.4%)	24 w	NR	NR	NR
Iborra [26]	49/407 (12%)	NR	Dose optimisation: 114/407 (28%) * Dose escalation: 12/407 (2.9%) * Intensification: 102/407 (25.1%)	NR	NR
Kubesch [27]	8/106 (7.5%)	NR	Intensification: 4/106 (3.8%)	NR	NR
Liefferinckx [28]	41/152 (27%)	NR	Intensification: 10/152 (6.6%)	NR	10/10 (100%)
Rajagopalan [30]	8/33 (24.2%) *	52 w	NR	NR	NR
Saman [31]	3/41 (7.3%)	NR	NR	NR	NR
Verstockt [33]	31/86 (36%)	24 w	NR	NR	NR
Af Björkesten [34]	17/155 (11%)	104 w	NR	NR	NR
Alric [35]	NR	NR	Intensification: 32/107 (30.1%)	58w	NR
Bar-Gil Shitrit [36]	7/106 (6.6%)	24 w	NR	NR	NR
Bennett [37]	23/96 (16.7%)	20.6 w	Intensification: 34/96 (35.4%)	NR	Endoscopic response: 7/14 (50%). Endoscopic remission: 5/14 (35.7%).
Biemans [38]	59/221 (26.7%)	24.6 w	Dose optimisation: 38/221 (17.2%) * Dose escalation: 31/221 (14%) * Intensification: 7/221 (3.2%)	48 w	CE free clinical remission * Dose escalation: 17/31 (54.8%) Clinical remission: * Intensification: 3/7 (42.9%)
Casas [40]	NR	NR	Intensification: 10/69 (14%)	NR	NR
Gadhok [41]	10/211 (4.7%)	NR	NR	NR	NR
Gubbiotti [42]	NR	NR	Dose escalation: 84/104 (80.9%)	32 w	NR
Harris [43]	7/84 (8.3%)	36.6 w	Intensification: 8/84 (9.5%)	NR	1/8 (12.5%)
Kakkadasam [44]	7/84 (8.3%)	36.6 w	22/76 (29%)	52.5 w	NR
Kopylov [45]	31/98 (31.6%)	26 w	Intensification: * q8w-q4w: 91/142 (64.1%) * q8w-q6w: 20/142 (14.1%) * IV reinduction: 14/142 (12%) * IV reinduction + interval shortening: 17/142 (12%)	29 w (mean)	Clinical response w16 from dose optimisation: 73/142 (51.4%) Clinical remission w16 from dose optimisation: 55/142 (38.7%) CE free clinical remission w16 from dose optimisation: 6/34 (17.6%) Endoscopic response 24w from dose optimisation: 10/23 (43.4%) Mucosal healing 24w from dose optimisation: 2/23 (8.6%) Clinical response w52 from dose optimisation: 51/98 (52%) Clinical remission w52 from dose optimisation: 41/98 (42%) CE free clinical remission w52 from dose optimisation: 9/34 (26.5%)
López-Tobaruela [46]	NR	NR	Intensification: 11/37 (29.7%) * q6w/q4w: 7/37 (18.9%) * IV reinduction: 4/37 (10.8%)	NR	NR
Monin [48]	17/118 (14.4%)	59.6 w	Intensification: 17/118 (14.4%)	NR	NR
Parra [51]	17/39 (43.6%)	NR	Intensification: * q8w-q4w: 8/245 (3.2%)	NR	4/8 (50%)
Saldaña [52]	11/35 (31.4%) 3/12 (25%)	24 w 52 w	Intensification: 6/35 (17%) *q8w-q4w: 4/35 (11.3%) * q8w-q6w: 2/35 (5.7%)	24 w	NR
Sánchez-Rodríguez [53]	0/25 (0%)	NR	NR	NR	NR
Tomasic [55]	5/42 (11.9%)	64 w (mean)	13/42 (31%)	NR	NR
Truyens [56]	3/67 (4.5%)	27.5 w	Intensification: 29/67 (43.3%) * IV reinduction: 2/67 (3%) * Shortening dosage interval: 16/67 (23.9%) * IV reinduction + shortening interval: 11/67 (16.4%)	NR	Clinical response: 15/22 (68.2%) Clinical remission: 5/22 (22.7%)
Cohen [59]	6/11 (54.5%)	2–24 m	Intensification: 9/11 (81.8%)	8–52 w	Clinical remission: 3/9 (33.3%)
Forss [60]	6/114 (5.3%)	16 w	NR	NR	NR
Garg [61]	(a) Elderly patients: 3/39 (7.7%) (b) Young patients: 9/78 (11.5%)	NR	Dose escalation: (a) Elderly patients: 7/39 (17.9%) (b) Young patients: 20/78 (25.6%)	NR	NR
Gonczi [62]	14/142 (9.9%)	60 w	Dose optimisation: 77/142 (54.2%) Dose escalation: 61/142 (43%) Intensification: 16/142 (11.2%)	NR	NR
Kim [63]	5/38 (13.2%)	62.1 w	Intensification: 18/38 (47.4%): * q8w-q4w: 15/38 (39.5%) * q8w-q6w: 1/38 (2.6%) * IV reinduction: 2/38 (5.3%)	NR	Clinical remission: 11/18 (61.1%)
Lorenzo [64]	12/98 (12.2%)	36 w (mean)	NR	NR	NR
Manlay [65]	NR	NR	Intensification: 96/224 (42.9%)	NR	NR
Plevris [67]	NR	NR	Dose optimisation: 30/216 (13.9%) * Dose escalation: 11/216 (5.1%) * Intensification q8w-q6w: 4/216 (1.9%) * Intensification q8w-q4w: 15/216 (6.9%)	NR	NR
Saiz [68]	7/49 (14.3%)	NR	16/49 (33%)	NR	NR
Sipponen [70]	15/155 (9.7%)	16 w	47/140: 33.6% * Dose escalation: 22/140 (15.7%) * Intensification: 25/140 (17.9%)	NR	41/47 (87.2%)
Straatmijer [71]	167/251 (66.7%)	52 w	NR	NR	NR
Tursi [72]	NR	NR	Intensification: 1/194 (0.5%)	NR	NR
Viola [73]	13/131 (9.9%)	52 w	NR	NR	NR
Chaparro [75]	13/456 (12.7%)	60 w	Dose optimisation: 121/463 (26.1%) * Dose escalation: 21/463 (4.5%) * Intensification: 100/463 (21.6%)	NR	Clinical remission: 63/80 (78.8%) * Dose escalation: 16/20 (80%) * Intensification (q8w-q4w): 42/54 (77.8%) * IV reinduction: 5/6 (83.3%)
Lenti [76]	65/259 (25%)	NR	NR	NR	NR

* Primary or secondary loss of response. ** Dose escalation: shortening of the administration interval from every 12 weeks to every 8 weeks; dose intensification: shortening of the ustekinumab administration interval to less than 8 weeks (every 4 or 6 weeks), or administration of a reinduction IV dose of ustekinumab. IV: intravenous; NR: not reported.

**Table 4:** Safety of ustekinumab.

Authors	AEs	Patients with AEs	AEs Requiring Ustekinumab Discontinuation	SAEs	Infections	Arthralgia or Myalgia	Skin Reactions	Infusion or Allergic Reaction	Headache	Malignancy	Others
Kopylov [14]	NR	NR	NR	NR	NR	NR	NR	NR	NR	NR	NR
Harris [15]	5/45 (11.1%)	5/45 (11.1%)	NR	NR	4/45 (8.9%)	0	1/45 (2.2%)	0	0	0	0
Khorrami [16]	14/116 (12.1%)	11/116 (9.5%)	0/116 (0%)	0/116 (0%)	3/116 (2.6%)	1/116 (0.8%)	1/116 (0.8%)	0/116 (0%)	2/116 (1.7%)	0/116 (0%)	4/116 (3.4%)
Battat [17]	43/62 (69.4%)	43/62 (69.4%)	2/62 (3.2%)	2/62 (3.2%)	3/62 (4.8%)	8/62 (12.9%)	5/62 (8.1%)	0/62 (0%)	14/62 (22.5%)	2/62 (3.2%)	11/62 (17.7%)
Greenup [18]	18/73 (24.7%)	18/73 (24.7%)	NR	NR	4/73 (5.5%)	6/73 (8.2%)	2/73 (2.7%)	1/73 (1.4%)	4/73 (5.5%)	0/73 (0%)	1/73 (1.4%)
Ma (a) [19]	61/167 (36.5%)	53/167 (31.1%)	11/167 (6.6%)	11/167 (6.6%)	20/167 (12%)	19/167 (11.4%)	3/167 (1.8%)	11/167 (6.6%)	6/167 (3.6%)	0/67 (0%)	2/167 (1.2%)
Ma (b) [20]	34/104 (32.7%)	34/104 (32.7%)	1/104 (1%)	NR	12/104 (11.5%)	13/104 (12.5%)	NR	NR	NR	NR	1/104 (1%)
Wils [21]	NR	NR	5/88 (5.7%)	1/88 (1.1%)	2/88 (2.2%)	1/88 (1.1%)	1/88 (1.1%)	NR	NR	1/88 (1.1%)	NR
Ahmed [22]	NR	NR	NR	NR	NR	NR	NR	NR	_	NR	NR
Calvo [23]	0/68 (0%)	0/68 (0%)	0/68 (0%)	0/68 (0%)	0/68 (0%)	0/68 (0%)	0/68 (0%)	0/68 (0%)	0/68 (0%)	0/68 (0%)	0/68 (0%)
Hernández-Camba [24]	1/47 (2.1%)	1/47 (2.1%)	NR	NR	1/47 (2.1%)	0/47 (0%)	0/47 (0%)	0/47 (0%)	0/47 (0%)	0/47 (0%)	0/47 (0%)
Hoffmann [25]	140/57 (245.6%)	140/57 (245.6%)	NR	NR	24/57 (42.1%)	24/57 (42.1%)	18/57 (31.6%)	0/57 (0%)	11/57 (19.3%)	0/57 (0%)	63/57 (110.5%)
Iborra [26]	71/407 (17.4%)	60/407 (14.7%)	NR	NR	40/407 (9.8%)	5/407 (1.2%)	5/407 (1.2%)	0/407 (0%)	2/407 (0.5%)	1/407 (0.25%)	18/407 (4.4%)
Kubesch [27]	3/106 (2.8%)	3/106 (2.8%)	0/106 (0%)	0/106 (0%)	2/106 (1.9%)	0/106 (0%)	0/106 (0%)	0/106 (0%)	0/106 (0%)	0/106 (0%)	1/106 (0.9%)
Liefferinckx [28]	11/152 (7.2%)	11/152 (7.2%)	1/152 (0.7%)		6/152 (3.9%)	2/152 (1.3%)	0/152 (0%)	1/152 (0.7%)	0/152 (0%)	0/152 (0%)	2/152 (1.3%)
Lynn [29]	56/594 (9.4%)	56/594 (9.4%)	NR	NR	51/594 (8.6%)	NR	NR	NR	NR	NR	5/594 (0.8%)
Rajagopalan [30]	3/33 (9%)	3/33 (9%)	NR	NR	1/33 (3%)	2/33 (6%)	0/33 (0%)	0/33 (0%)	0/33 (0%)	0/33 (0%)	0/33 (0%)
Saman [31]	2/41 (4.8%)	2/41 (4.8%)	2/41 (4.8%)	0/41 (0%)	0/41 (0%)	1/41 (2.4%)	1/41 (2.4%)	0/41 (0%)	0/41 (0%)	0/41 (0%)	0/41 (0%)
Townsend [32]	NR	NR	7/45 (15.6%)	NR	NR	NR	NR	NR	NR	NR	NR
Verstockt [33]	12/86 (14%)	12/86 (14%)	5/86 (5.8%)	NR	6/86 (7%)	2/86 (2.4%)	1/86 (1.2%)	0/86 (0%)	0/86 (0%)	0/86 (0%)	3/86 (3.5%)
af Björkesten [34]	5/155 (3.2%)	NR	5/155 (3.2%)	NR	3/155 (1.9%)	0/155 (0%)	0/155 (0%)	0/155 (0%)	0/155 (0%)	1/155 (0.6%)	1/155 (0.6%)
Alric [35]	25/107 (23.4%)	21/107 (19.6%)	1/107 (0.9%)	NR	12/107 (11.2%)	1/107 (0.9%)	6/107 (5.6%)	0/107 (0%)	1/107 (0.9%)	1/107 (0.9%)	4/107 (3.7%)
Bar-Gil Shitrit [36]	15/106 (14.2%)	15/106 (14.2%)	3/106 (2.8%)	0/106 (0%)	0/106 (0%)	4/106 (3.6%)	3/106 (2.8%)	0/106 (0%)	1/106 (0.9%)	0/106 (0%)	7/106 (6.6%)
Bennett [37]	5/96 (5.2%)	5/96 (5.2%)	NR	NR	NR	NR	NR	NR	NR	NR	NR
Biemans [38]	110/221 (49.8%)	110/221(49.8%)	8/221 (3.6%)	NR	70/221 (31.7%):	5/221 (2.3%)	13/221 (5.9%)	1/221 (0.4%)	7/221 (3.2%)	0/221 (0%)	14/221 (6.3%)
Calvo [39]	NR	NR	NR	NR	NR	NR	NR	NR	NR	NR	NR
Casas [40]	4/69 (5.8%)	4/69 (5.8%)	0/69 (0%)	0/69 (0%)	0/69 (0%)	1/69 (1.4%)	2/69 (2.9%)	0/69 (0%)	0/69 (0%)	0/69 (0%)	1/69 (1.4%)
Gadhok [41]	27/211 (12.8%)	27/211 (12.8%)	NR	NR	NR	NR	NR	NR	NR	NR	NR
Gubbiotti [42]	3/104 (2.9%)	3/104 (2.9%)	2/104 (1.9%)	NR	NR	NR	NR	NR	NR	NR	NR
Harris [43]	21/84 (25%)	21/84 (25%)	3/84 (3.6%)	20/84 (23.8%)	4/84 (4.8%)	0/84 (0%)	1/84 (1.2%)	0/84 (0%)	0/84 (0%)	0/84 (0%)	16/84 (19%)
Kakkadasam [44]	1/76 (1.3%)	1/76 (1.3%)	1/76 (1.3%)	0/76 (0%)	0/76 (0%)	1/76 (1.3%)	0/76 (0%)	0/76 (0%)	0/76 (0%)	0/76 (0%)	0/76 (0%)
Kopylov [45]	11/142 (7.7%)	11/142 (7.7%)	1/142 (0.7%)	1/142 (0.7%)	5/142 (3.5%)	0/142 (0%)	2/142 (1.4%)	0/142 (0%)	0/142 (0%)	2 (1.4%)	2 (1.4%)
López-Tobaruela [46]	3/37 (8.1%)	3/37 (8.1%)	NR	NR	NR	NR	NR	NR	NR	NR	NR
Mohammad [47]	16/123 (13%)	16/123 (13%)	NR	NR	13/123 (1%)	NR	NR	NR	NR	NR	NR
Monin [48]	26/156 (17.1%)	26/156 (17.1%)	9/156 (5.8%)	7/156 (4.6%)	26/156 (17.1%)	NR	NR	NR	NR	NR	NR
Mozdiak [49]	8/62 (13%)	8/62 (13%)	NR	3/62 (4.8%)	NR	NR	NR	NR	NR	NR	NR
Rayer [50]	NR	NR	NR	NR	NR	NR	NR	NR	NR	NR	NR
Parra [51]	60/245 (24.5%)	48/245 (19.6%)	8/245 (3.2%)	8/245 (3.2%)	14/245 (5.7%)	2/245 (0.8%)	9/245 (3.7%)	NR	3/245 (1.2%)	0/245 (0%)	NR
Saldaña [52]	NR	NR	2/61 (3.3%)	11/61 (18%)	1/61 (1.6%)	NR	NR	NR	NR	NR	10/61 (16.4%)
Sánchez-Rodríguez [53]	NR	NR	NR	NR	NR	NR	NR	NR	NR	NR	NR
Shim [54]	NR	NR	NR	NR	NR	NR	NR	0/22 (0%)	NR	0/22 (0%)	NR
Tomasic [55]	NR	NR	NR	NR	NR	NR	NR	NR	NR	NR	NR
Truyens [56]	2/67 (3%)	2/67 (3%)	2/67 (3%)	NR	0/67 (0%)	0/67 (0%)	0/67 (0%)	0/67 (0%)	0/67 (0%)	1/67 (1.5%)	1/67 (1.5%)
Bokemeyer [57]	NR	NR	NR	NR	NR	NR	NR	NR	NR	NR	NR
Casas [58]	79/648 (12.2%) * Elderly: 30/212 (14.2%) * Young: 49/436 (11.2%)	79/648 (12.2%)	47/648 (7.3%)	NR	NR	NR	NR	NR	NR	* Elderly: 9/212 (4.3%) * Young: 3/436 (0.69%)	NR
Cohen [59]	0/11 (0%)	0/11 (0%)	0/11 (0%)	0/11 (0%)	0/11 (0%)	0/11 (0%)	0/11 (0%)	0/11 (0%)	0/11 (0%)	0/11 (0%)	0/11 (0%)
Forss [60]	NR	NR	NR	0/114 (0%)	NR	NR	NR	NR	NR	NR	NR
Garg [61]	14/117 (12%) * Elderly: 3/39 (7.7%) * Young: 11/78 (14.1%)	14/117 (12%)	3/117 (2.6%)	NR	8/117 (6.8%) * Elderly: 2/39 (5.2%) * Young: 6/78 (7.7%)	0/117 (0%)	0/117 (0%)	6/117 (5.1%) * Elderly: 1/39 (2.5%) * Young: 5/78 (6.4%)	0/117 (0%)	0/117 (0%)	0/117 (0%)
Gonczi [62]	5/142 (3.5%)	5/142 (3.5%)	2/142 (1.4%)	0/142 (0%)	0/142 (0%)	1/142 (0.7%)	4/142 (2.8%)	0/142 (0%)	0/142 (0%)	0/142 (0%)	0/142 (0%)
Kim [63]	4/38 (10.5%):	4/38 (10.5%):	0/38 (0%)	1/38 (2.6%)	3/38 (7.9%)	0/38 (0%)	0/38 (0%)	1/38 (2.6%)	0/38 (0%)	0/38 (0%)	0/38 (0%)
Lorenzo [64]	4/98 (4%):	4/98 (4%):	0/98 (0%)	0/98 (0%)	1/98 (1%)	0/98 (0%)	1/98 (1%)	1/98 (1%)	0/98 (0%)	0/98 (0%)	1/98 (1%)
Manlay [65]	NR	NR	11/224 (4.9%)	NR	NR	NR	NR	NR	NR	NR	NR
Miranda [66]	NR	NR	NR	0/92 (0%)	NR	NR	NR	NR	NR	NR	NR
Plevris [67]	NR	NR	NR	19/216 (8.8%)	NR	NR	NR	NR	NR	NR	NR
Saiz [68]	NR	NR	NR	0/49 (0%)	NR	NR	NR	NR	NR	NR	NR
Scribano [69]	10/140 (7.1%)	9/140 (6.4%)	3/140 (2.1%)	NR	0/140 (0%)	1/140 (0.7%)	3/140 (2.1%)	2/140 (1.4%)	1/140 (0.7%)	0/140 (0%)	3/140 (2.1%)
Sipponen [70]	NR	NR	NR	NR	NR	NR	NR	NR	NR	NR	NR
Straatmijer [71]	NR	NR	8/252 (3.2%)	NR	NR	NR	NR	NR	NR	2/252 (7.9%)	NR
Tursi [72]	5/194 (2.6%)	5/194 (2.6%)	4/194 (2%)	0/194 (0%)	1/194 (0.5%)	2/194 (1%)	0/194 (0%)	1/194 (0.5%)	0/194 (0%)	0/194 (0%)	1/194 (0.5%)
Viola [73]	21/131 (16%)	21/131 (16%)	3/131 (2.1%)	0/131 (0%)	4/131 (3%)	1/131 (0.7%)	2/131 (1.4%)	0/131 (0%)	0/131 (0%)	0/131 (0%)	14/131 (10.7%)
Yokoyama [74]	24/339 (7.1%)	18/339 (5.3%)	NR	7/339 (2.1%)	NR	NR	NR	NR	NR	NR	NR
Chaparro [75]	50/463 (10.8%)	39/463 (8.4%)	4/463 (0.9%)	4/463 (0.9%)	5/463 (1.1%)	5/463 (1.1%)	9/463 (1.9%)	38/463 (8.2%)	7/463 (1.5%)	1/463 (0.2%)	13/463 (2.8%)
Lenti [76]	130/256 (50.2%)	130/256 (50.2%)	NR	103 (40.2%)	NR	8/256 (3.1%)	6/256 (2.3%)	NR	8/256 (3.1%)	3/256 (1.1%)	5/256 (1.9%)

* AEs: adverse events; SAEs: serious adverse events; NR: not reported.

**Table 5:** Newcastle–Ottawa scale for assessment of quality of included studies (each asterisk represents when an individual criterion within the subsection was fulfilled, See Supplementary Figure S1).

Authors	Selection				Comparability	Outcome			
	Representativeness of The Exposed Cohort	Selection of the Non-Exposed Cohort?	Ascertainment of Exposure	Demonstration that Outcome of Interest Was Not Present at the Start of the Study?	Comparability of Cohorts on the Basis of the Design or Analysis	Assessment of Outcome?	Was Follow-Up Long Enough for Outcome to Occur?	Adequacy of Follow-Up of Cohorts?	Overall Quality Score (Max. = 9)
Kopylov [14]	*		*	*		*	*		5
Harris [15]	*		*	*		*	*	*	6
Khorrami [16]	*		*	*		*	*	*	6
Battat [17]	*		*			*	*	*	5
Greenup [18]	*		*	*		*	*	*	6
Ma (a) [19]	*		*	*		*	*	*	6
Ma (b) [20]	*		*	*		*	*	*	6
Wils [21]	*		*	*		*	*	*	6
Ahmed [22]	*	*	*	*	*	*	*	*	8
Hoffmann [25]	*		*	*		*	*	*	6
Iborra [26]	*		*	*		*	*	*	6
Kubesch [27]	*		*	*		*	*		5
Liefferinckx [28]	*		*	*		*	*	*	6
Saman [31]	*		*	*		*	*	*	6
Townsend [32]	*	*	*	*	**	*	*	*	9
Verstockt [33]	*		*	*		*	*	*	6
af Björkesten [34]	*		*	*		*	*	*	6
Alric [35]	*	*	*	*	**	*	*	*	9
Bar-Gil Shitrit [36]	*		*	*		*	*	*	6
Bennett [37]	*		*	*		*	*	*	6
Biemans [38]	*		*	*		*	*		5
Casas [40]	*		*	*		*	*		5
Harris [43]	*		*	*		*	*	*	6
Kopylov [45]	*		*	*		*	*		5
Monin [48]	*		*	*		*	*	*	6
Parra [51]	*		*	*		*	*	*	6
Saldaña [52]	*		*	*		*	*	*	6
Cohen [59]	*		*	*		*	*	*	6
Forss [60]	*		*	*		*	*	*	6
Garg [61]	*	*	*	*	*	*	*	*	8
Gonczi [62]	*		*	*		*	*	*	6
Kim [63]	*		*	*		*	*	*	6
Lorenzo [64]	*		*	*		*	*		5
Manlay [65]	*	*	*	*	*	*	*	*	8
Miranda [66]	*		*	*		*	*		5
Plevris [67]	*		*	*		*	*	*	6
Scribano [69]	*		*	*		*	*	*	6
Sipponen [70]	*		*	*		*	*	*	6
Straatmijer [71]	*		*	*		*	*		5
Tursi [72]	*		*	*		*	*	*	6
Viola [73]	*		*	*		*	*		5
Yokoyama [74]	*		*	*		*	*		6
Chaparro [75]	*		*	*		*	*	*	6

## Data Availability

Data sharing not applicable.

## Supplementary Materials

The following supporting information can be downloaded at: https://www.mdpi.com/article/10.3390/jcm11144202/s1, Figure S1: Newcastle–Ottawa quality assessment scale; Figure S2: Corticosteroid-free clinical remission; Figure S3: Endoscopic remission; Figure S4: Publication bias.

## References

[1] Wilson J.C., Furlano R.I., Jick S.S., Meier C.R. (2016). Inflammatory Bowel Disease and the Risk of Autoimmune Diseases. J. Crohn’s Colitis.

[2] Torres J., Bonovas S., Doherty G., Kucharzik T., Gisbert J.P., Raine T., Adamina M., Armuzzi A., Bachmann O., Bager P. (2020). ECCO Guidelines on Therapeutics in Crohn’s Disease: Medical Treatment. J. Crohn’s Colitis.

[3] Gisbert J.P., Chaparro M. (2021). Primary Failure to an Anti-TNF Agent in Inflammatory Bowel Disease: Switch (to a Second Anti-TNF Agent) or Swap (for Another Mechanism of Action)?. J. Clin. Med..

[4] Gisbert J.P., Chaparro M. (2020). Predictors of Primary Response to Biologic Treatment [Anti-TNF, Vedolizumab, and Ustekinumab] in Patients with Inflammatory Bowel Disease: From Basic Science to Clinical Practice. J. Crohn’s Colitis.

[5] Colombel J.F., Feagan B.G., Sandborn W.J., Van Assche G., Robinson A.M. (2012). Therapeutic drug monitoring of biologics for inflammatory bowel disease. Inflamm. Bowel Dis..

[6] Feagan B.G., Sandborn W.J., Gasink C., Jacobstein D., Lang Y., Friedman J.R., Blank M.A., Johanns J., Gao L.L., Miao Y. (2016). Ustekinumab as Induction and Maintenance Therapy for Crohn’s Disease. N. Engl. J. Med..

[7] Hanauer S.B., Sandborn W.J., Feagan B.G., Gasink C., Jacobstein D., Zou B., Johanns J., Adedokun O.J., Sands B.E., Rutgeerts P. (2020). IM-UNITI: Three-year Efficacy, Safety, and Immunogenicity of Ustekinumab Treatment of Crohn’s Disease. J. Crohn’s Colitis.

[8] Sandborn W.J., Rebuck R., Wang Y., Zou B., Adedokun O.J., Gasink C., Sands B.E., Hanauer S.B., Targan S., Ghosh S. (2022). Five-Year Efficacy and Safety of Ustekinumab Treatment in Crohn’s Disease: The IM-UNITI Trial. Clin. Gastroenterol. Hepatol..

[9] Adamina M., Bonovas S., Raine T., Spinelli A., Warusavitarne J., Armuzzi A., Bachmann O., Bager P., Biancone L., Bokemeyer B. (2020). ECCO Guidelines on Therapeutics in Crohn’s Disease: Surgical Treatment. J. Crohn’s Colitis.

[10] Gisbert J.P., Chaparro M. (2017). Ustekinumab to treat Crohn’s disease. Gastroenterol. Hepatol..

[11] Moher D., Liberati A., Tetzlaff J., Altman D.G., Group P. (2009). Preferred reporting items for systematic reviews and meta-analyses: The PRISMA statement. BMJ.

[12] Stroup D.F., Berlin J.A., Morton S.C., Olkin I., Williamson G.D., Rennie D., Moher D., Becker B.J., Sipe T.A., Thacker S.B. (2000). Meta-analysis of observational studies in epidemiology: A proposal for reporting. Meta-analysis of Observational Studies in Epidemiology (MOOSE) group. JAMA.

[13] Begg C.B., Mazumdar M. (1994). Operating characteristics of a rank correlation test for publication bias. Biometrics.

[14] Kopylov U., Afif W., Cohen A., Bitton A., Wild G., Bessissow T., Wyse J., Al-Taweel T., Szilagyi A., Seidman E. (2014). Subcutaneous ustekinumab for the treatment of anti-TNF resistant Crohn’s disease—The McGill experience. J. Crohn’s Colitis.

[15] Harris K.A., Horst S., Gadani A., Nohl A., Annis K., Duley C., Beaulieu D., Ghazi L., Schwartz D.A. (2016). Patients with Refractory Crohn’s Disease Successfully Treated with Ustekinumab. Inflamm. Bowel Dis..

[16] Khorrami S., Ginard D., Marin-Jimenez I., Chaparro M., Sierra M., Aguas M., Sicilia B., Garcia-Sanchez V., Suarez C., Villoria A. (2016). Ustekinumab for the Treatment of Refractory Crohn’s Disease: The Spanish Experience in a Large Multicentre Open-label Cohort. Inflamm. Bowel Dis..

[17] Battat R., Kopylov U., Bessissow T., Bitton A., Cohen A., Jain A., Martel M., Seidman E., Afif W. (2017). Association between Ustekinumab trough Concentrations and Clinical, Biomarker, and Endoscopic Outcomes in Patients with Crohn’s Disease. Clin. Gastroenterol. Hepatol..

[18] Greenup A.J., Rosenfeld G., Bressler B. (2017). Ustekinumab use in Crohn’s disease: A Canadian tertiary care centre experience. Scand. J. Gastroenterol..

[19] Ma C., Fedorak R.N., Kaplan G.G., Dieleman L.A., Devlin S.M., Stern N., Kroeker K.I., Seow C.H., Leung Y., Novak K.L. (2017). Clinical, endoscopic and radiographic outcomes with ustekinumab in medically-refractory Crohn’s disease: Real world experience from a multicentre cohort. Aliment. Pharmacol. Ther..

[20] Ma C., Fedorak R.N., Kaplan G.G., Dieleman L.A., Devlin S.M., Stern N., Kroeker K.I., Seow C.H., Leung Y., Novak K.L. (2017). Long-term Maintenance of Clinical, Endoscopic, and Radiographic Response to Ustekinumab in Moderate-to-Severe Crohn’s Disease: Real-world Experience from a Multicenter Cohort Study. Inflamm. Bowel Dis..

[21] Wils P., Bouhnik Y., Michetti P., Flourie B., Brixi H., Bourrier A., Allez M., Duclos B., Serrero M., Buisson A. (2018). Long-term efficacy and safety of ustekinumab in 122 refractory Crohn’s disease patients: A multicentre experience. Aliment. Pharmacol. Ther..

[22] Ahmed Z., Venkata K., Zhang N., Malik T.A. (2019). Comparative Effectiveness of Ustekinumab versus Adalimumab in Induction of Clinical Response and Remission in Crohn’s Disease: Experience of a Real-World Cohort at a Tertiary Care Inflammatory Bowel Disease Referral Center. Gastroenterol. Res..

[23] Gonzalez-Lama Y., Bermejo F., Lopez-Sanroman A., Garcia-Sanchez V., Esteve M., Cabriada J.L., McNicholl A.G., Pajares R., Casellas F., Merino O. (2011). Thiopurine methyl-transferase activity and azathioprine metabolite concentrations do not predict clinical outcome in thiopurine-treated inflammatory bowel disease patients. Aliment. Pharmacol. Ther..

[24] Hernandez-Camba A., Arranz L., Vera I., Carpio D., Calafat M., Lucendo A.J., Taxonera C., Marin S., Garcia M.J., Marin G.S. (2021). Real-world use of mycophenolate mofetil in inflammatory bowel disease: Results from the ENEIDA registry. Dig. Liver Dis..

[25] Hoffmann P., Krisam J., Wehling C., Kloeters-Plachky P., Leopold Y., Belling N., Gauss A. (2019). Ustekinumab: “Real-world” outcomes and potential predictors of nonresponse in treatment-refractory Crohn’s disease. World J. Gastroenterol..

[26] Iborra M., Beltran B., Fernandez-Clotet A., Iglesias-Flores E., Navarro P., Rivero M., Gutierrez A., Sierra-Ausin M., Mesonero F., Ferreiro-Iglesias R. (2020). Real-world long-term effectiveness of ustekinumab in Crohn’s disease: Results from the ENEIDA registry. Aliment. Pharmacol. Ther..

[27] Kubesch A., Rueter L., Farrag K., Krause T., Stienecker K., Hausmann J., Filmann N., Dignass A., Stein J., Blumenstein I. (2019). Short and Long-Term Effectiveness of Ustekinumab in Patients with Crohn’s Disease: Real-World Data from a German IBD Cohort. J. Clin. Med..

[28] Liefferinckx C., Verstockt B., Gils A., Noman M., Van Kemseke C., Macken E., De Vos M., Van Moerkercke W., Rahier J.F., Bossuyt P. (2019). Long-term Clinical Effectiveness of Ustekinumab in Patients with Crohn’s Disease Who Failed Biologic Therapies: A National Cohort Study. J. Crohn’s Colitis.

[29] Lynn A.M., Loftus E.V. (2018). Illuminating the Black Box: The Real Risk of Serious Infection with Inflammatory Bowel Disease Therapies. Gastroenterology.

[30] Rajagopalan A.N., Chaudhuri S., Mudenagudi U. (2004). Depth estimation and image restoration using defocused stereo pairs. IEEE Trans. Pattern Anal. Mach. Intell..

[31] Saman S., Goetz M., Wendler J., Malek N.P., Wehkamp J., Klag T. (2019). Ustekinumab is effective in biological refractory Crohn’s disease patients-regardless of approval study selection criteria. Intest. Res..

[32] Townsend T., Razanskaite V., Dodd S., Storey D., Michail S., Morgan J., Davies M., Penman D., Watters C., Swaminathan M. (2020). Comparative effectiveness of ustekinumab or vedolizumab after one year in 130 patients with anti-TNF-refractory Crohn’s disease. Aliment. Pharmacol. Ther..

[33] Verstockt B., Dreesen E., Noman M., Outtier A., Van den Berghe N., Aerden I., Compernolle G., Van Assche G., Gils A., Vermeire S. (2019). Ustekinumab Exposure-outcome Analysis in Crohn’s Disease Only in Part Explains Limited Endoscopic Remission Rates. J. Crohn’s Colitis.

[34] Af Bjorkesten C.G., Ilus T., Hallinen T., Soini E., Eberl A., Hakala K., Heikura M., Jussila A., Koskela R., Koskinen I. (2020). Objectively assessed disease activity and drug persistence during ustekinumab treatment in a nationwide real-world Crohn’s disease cohort. Eur. J. Gastroenterol. Hepatol..

[35] Alric H., Amiot A., Kirchgesner J., Treton X., Allez M., Bouhnik Y., Beaugerie L., Carbonnel F., Meyer A. (2020). The effectiveness of either ustekinumab or vedolizumab in 239 patients with Crohn’s disease refractory to anti-tumour necrosis factor. Aliment. Pharmacol. Ther..

[36] Bar-Gil Shitrit A., Ben-Ya’acov A., Siterman M., Waterman M., Hirsh A., Schwartz D., Zittan E., Adler Y., Koslowsky B., Avni-Biron I. (2020). Safety and effectiveness of ustekinumab for induction of remission in patients with Crohn’s disease: A multicenter Israeli study. United Eur. Gastroenterol. J..

[37] Bennett A., Evers Carlini L., Duley C., Garrett A., Annis K., Wagnon J., Dalal R., Scoville E., Beaulieu D., Schwartz D. (2020). A Single Center Experience with Long-Term Ustekinumab Use and Reinduction in Patients with Refractory Crohn Disease. Crohn’s Colitis 360.

[38] Biemans V.B.C., van der Meulen-de Jong A.E., van der Woude C.J., Lowenberg M., Dijkstra G., Oldenburg B., de Boer N.K.H., van der Marel S., Bodelier A.G.L., Jansen J.M. (2020). Ustekinumab for Crohn’s Disease: Results of the ICC Registry, a Nationwide Prospective Observational Cohort Study. J. Crohn’s Colitis.

[39] Calvo Moya M.I., Omella Usieto I., González Lama Y., Matallana Royo V., González Partida I., Menchen Viso B., De Lucas Téllez de Meneses R., González Rodriguez M., Bella del Castillo P., Vera Mendoza M.I. (2020). P545 Deep remission assessed by endoscopy, magnetic resonance or intestinal ultrasound, in refractory Crohn’s disease patients in clinical remission with ustekinumab: A real-life single-centre experience. J. Crohn’s Colitis.

[40] Casas Deza D., Garcia Lopez S., Lafuente Blasco M., Vicente Lidon R., Nerin de la Puerta J., Pena Gonzalez E., Ber Nieto Y., Charro Calvillo M., Alcala Escriche M.J., Gomollon Garcia F. (2020). Efficacy and safety of ustekinumab in real clinical practice. Retrospective multicentre study. ARAINF cohort. Gastroenterol. Hepatol..

[41] Gadhok R., Fragkos K., Honap S., Hassan J., Whiteley L., Ibarra A., Burgess N., Vega R., Seward E., Mehta S. (2020). P507 Ustekinumab: Medium-term outcomes from a UK multicentre real-world cohort. J. Crohn’s Colitis.

[42] Barberio B., Savarino E.V., Card T., Canova C., Baldisser F., Gubbiotti A., Massimi D., Ghisa M., Zingone F. (2022). Incidence comparison of adverse events in patients with inflammatory bowel disease receiving different biologic agents: Retrospective long-term evaluation. Intest. Res..

[43] Harris R.J., McDonnell M., Young D., Bettey M., Downey L., Pigott L., Felwick R., Gwiggner M., Cummings J.R.F. (2020). Early real-world effectiveness of ustekinumab for Crohn’s disease. Frontline Gastroenterol..

[44] Kakkadasam Ramaswamy P., Moattar H., Sawyer E., Edwards J., Shukla D. (2020). P697 Efficacy and safety of ustekinumab in Crohn’s disease: A real-world study from Australia. J. Crohn’s Colitis.

[45] Kopylov U., Hanzel J., Liefferinckx C., De Marco D., Imperatore N., Plevris N., Baston-Rey I., Harris R.J., Truyens M., Domislovic V. (2020). Effectiveness of ustekinumab dose escalation in Crohn’s disease patients with insufficient response to standard-dose subcutaneous maintenance therapy. Aliment. Pharmacol. Ther..

[46] Lopez Tobaruela J.M., Sanchez-Capilla A.D., Ortega-Suazo E.J., Fernandez-Cano M.C., Herrador-Paredes M., Cabello-Tapia M.J., Martin-Rodriguez M.M. (2020). P577 Ustekinumab in actual clinical practice: Our centre experience. J. Crohn’s Colitis.

[47] Mohammad D., Alshahrani A., Bao Y., Alramdan R., Rajani A., Chauhan U., Salena B., Tse F., Greenwald E., Albashir S. (2020). P484 Effectiveness and safety of ustekinumab in patients with Crohn’s disease: A real-world experience. J. Crohn’s Colitis.

[48] Monin L., Dubois S., Reenaers C., Van Kemseke C., Latour P., Van Daele D., Vieujean S., Seidel L., Louis E. (2021). Ustekinumab in bio-naive and bio-failure Crohn’s disease patients: Results from a <<real-life>> monocentric cohort. Dig. Liver Dis..

[49] Mozdiak E., Wicaksono A.N., Covington J.A., Arasaradnam R.P. (2019). Colorectal cancer and adenoma screening using urinary volatile organic compound (VOC) detection: Early results from a single-centre bowel screening population (UK BCSP). Tech. Coloproctol..

[50] Rayer C., Roblin X., Laharie D., Caron B., Flamant M., Dewitte M., Fumery M., Viennot S., Bourreille A., Pariente B. (2020). P665 Which second-line biologic after anti-TNF failure during Crohn’s disease: Ustekinumab or vedolizumab, a multicentre retrospective study. J. Crohn’s Colitis.

[51] Parra R.S., Chebli J.M.F., Queiroz N.S.F., Damiao A., de Azevedo M.F.C., Chebli L.A., Bertges E.R., Alves Junior A.J.T., Ambrogini Junior O., da Silva B. (2022). Long-term effectiveness and safety of ustekinumab in bio-naive and bio-experienced anti-tumor necrosis factor patients with Crohn’s disease: A real-world multicenter Brazilian study. BMC Gastroenterol..

[52] Saldana Duenas C., Rullan Iriarte M., Elosua Gonzalez A., Rodriguez Gutierrez C., Rubio Iturria S., Nantes Castillejo O. (2020). Ustekinumab in Crohn’s disease: Effectiveness and safety in clinical practice. Gastroenterol. Hepatol..

[53] Sánchez Rodríguez E., Mesonero Gismero F., López Sanroman A. (2020). P737 Ustekinumab induction effectiveness in Crohn’s disease in a real-life cohort. J. Crohn’s Colitis.

[54] Shim H.H., Kong S.C., Ong W.C., Lim T.G., Chan P.W. (2020). P381 Use of ustekinumab in Crohn’s disease: Singapore largest single-centre experience. J. Crohn’s Colitis.

[55] Duvnjak M., Bilic A., Barsic N., Tomasic V., Stojsavljevic S. (2013). Classical medications in the treatment of inflammatory bowel diseases. Acta Med. Croat..

[56] Truyens M., Geldof J., Dewitte G., Glorieus E., Hindryckx P., Lobaton Ortega T. (2020). P344 Effectiveness of ustekinumab in refractory Crohn’s disease: A real-life experience in a tertiary referral centre. J. Crohn’s Colitis.

[57] Bokemeyer B., Plachta-Danielzik S., Di Giuseppe R., Mohl W., Teich N., Hoffstadt M., Schweitzer A., von der Ohe M., Gauss A., Atreya R. (2021). DOP47 Real World Evidence on the effectiveness of ustekinumab in Crohn’s Disease: Induction phase results from the prospective, observational RUN-CD Study. J. Crohn’s Colitis.

[58] Casas Deza D., Lamuela Calvo L.J., Arbonés Mainar J.M., Ricart E., Gisbert J.P., Rivero Tirado M., Sanchez Rodríguez E., Sicilia B., Gutierrez Casbas A., Merino Ochoa O. (2021). P262 Effectiveness and safety of ustekinumab in elderly patients: Real world evidence from ENEIDA registry. J. Crohn’s Colitis.

[59] Cohen A., Ahmed N., Sant’Anna A. (2021). Ustekinumab for the treatment of refractory pediatric Crohn’s disease: A single-center experience. Intest. Res..

[60] Forss A., Clements M., Myrelid P., Strid H., Soderman C., Wagner A., Andersson D., Hjelm F., Olen O., The PROSE SWIBREG Study Group (2021). Prospective observational study on Stelara (ustekinumab) assessing effectiveness in Crohn’s disease (PROSE): A 16-week follow-up. Scand. J. Gastroenterol..

[61] Garg R., Aggarwal M., Butler R., Achkar J.P., Lashner B., Philpott J., Cohen B., Qazi T., Rieder F., Regueiro M. (2022). Real-World Effectiveness and Safety of Ustekinumab in Elderly Crohn’s Disease Patients. Dig. Dis. Sci..

[62] Gonczi L., Szanto K., Farkas K., Molnar T., Szamosi T., Schafer E., Golovics P.A., Barkai L., Lontai L., Lovasz B. (2022). Clinical efficacy, drug sustainability and serum drug levels in Crohn’s disease patients treated with ustekinumab—A prospective, multicenter cohort from Hungary. Dig. Liver Dis..

[63] Kim F.S., Patel P.V., Stekol E., Ali S., Hamandi H., Heyman M.B., Verstraete S.G. (2021). Experience Using Ustekinumab in Pediatric Patients with Medically Refractory Crohn Disease. J. Pediatr. Gastroenterol. Nutr..

[64] Lorenzo Gonzalez L., Valdes Delgado T., Vazquez Moron J.M., Castro Laria L., Leo Carnerero E., Maldonado Perez M.B., Sanchez Capilla D., Pallares Manrique H., Saez Diaz A., Arguelles Arias F. (2022). Ustekinumab in Crohn’s disease: Real-world outcomes and predictors of response. Rev. Esp. Enferm. Dig..

[65] Manlay L., Boschetti G., Pereira B., Flourie B., Dapoigny M., Reymond M., Sollelis E., Gay C., Boube M., Buisson A. (2021). Comparison of short- and long-term effectiveness between ustekinumab and vedolizumab in patients with Crohn’s disease refractory to anti-tumour necrosis factor therapy. Aliment. Pharmacol. Ther..

[66] Miranda A., Gravina A.G., Cuomo A., Mucherino C., Sgambato D., Facchiano A., Granata L., Priadko K., Pellegrino R., de Filippo F.R. (2021). Efficacy of ustekinumab in the treatment of patients with Crohn’s disease with failure to previous conventional or biologic therapy: A prospective observational real-life study. J. Physiol. Pharmacol..

[67] Plevris N., Fulforth J., Siakavellas S., Robertson A., Hall R., Tyler A., Jenkinson P.W., Campbell I., Chuah C.S., Kane C. (2021). Real-world effectiveness and safety of ustekinumab for the treatment of Crohn’s disease: The Scottish ustekinumab cohort. J. Gastroenterol. Hepatol..

[68] Saiz Chumillas R.M., Alba Hernández L., Chivato Martín-Falquina I., Badia Aranda E., Arias García M.L., Sicilia Aladrén B. (2021). P389 Efficacy and safety of ustekinumab in patients with Crohn’s disease refractory to anti-tumour necrosis factor: Real clinical practice. J. Crohn’s Colitis.

[69] Scribano M.L., Aratari A., Neri B., Bezzio C., Balestrieri P., Baccolini V., Falasco G., Camastra C., Pantanella P., Monterubbianesi R. (2022). Effectiveness of ustekinumab in patients with refractory Crohn’s disease: A multicentre real-life study in Italy. Ther. Adv. Gastroenterol..

[70] Sipponen T., Af Bjorkesten C.G., Hallinen T., Ilus T., Soini E., Eberl A., Heikura M., Kellokumpu M., Koskela R., Nielsen C. (2021). A nationwide real-world study on dynamic ustekinumab dosing and concomitant medication use among Crohn’s disease patients in Finland. Scand. J. Gastroenterol..

[71] Straatmijer T., Biemans V.B.C., Hoentjen F., de Boer N.K.H., Bodelier A.G.L., Dijkstra G., van Dop W.A., Haans J.J.L., Jansen J.M., Maljaars P.W.J. (2021). Ustekinuma b for Crohn’s Disease: Two-Year Results of the Initiative on Crohn and Colitis (ICC) Registry, a Nationwide Prospective Observational Cohort Study. J. Crohn’s Colitis.

[72] Tursi A., Mocci G., Cuomo A., Allegretta L., Aragona G., Colucci R., Della Valle N., Ferronato A., Forti G., Gaiani F. (2021). Real-life efficacy and safety of Ustekinumab as second- or third-line therapy in Crohn’s disease: Results from a large Italian cohort study. Eur. Rev. Med. Pharmacol. Sci..

[73] Viola A., Muscianisi M., Macaluso F.S., Ventimiglia M., Cappello M., Privitera A.C., Magnano A., Pluchino D., Magri G., Ferracane C. (2021). Ustekinumab in Crohn’s disease: Real-world outcomes from the Sicilian network for inflammatory bowel diseases. JGH Open.

[74] Yokoyama S., Asano T., Nagano K., Tsuchiya H., Takagishi M., Tsujioka S., Miura N., Matsumoto T. (2021). Safety and effectiveness of ustekinumab in Crohn’s disease: Interim results of post-marketing surveillance in Japan. J. Gastroenterol. Hepatol..

[75] Chaparro M., Baston-Rey I., Fernandez-Salgado E., Gonzalez Garcia J., Ramos L., Diz-Lois Palomares M.T., Arguelles-Arias F., Iglesias Flores E., Cabello M., Rubio Iturria S. (2022). Long-Term Real-World Effectiveness and Safety of Ustekinumab in Crohn’s Disease Patients: The SUSTAIN Study. Inflamm. Bowel Dis..

[76] Lenti M.V., Dolby V., Clark T., Hall V., Tattersall S., Fairhurst F., Kenneth C., Walker R., Kemp K., Borg-Bartolo S. (2022). A propensity score-matched, real-world comparison of ustekinumab vs. vedolizumab as a second-line treatment for Crohn’s disease. The Cross Pennine study II. Aliment. Pharmacol. Ther..

[77] Macaluso F.S., Maida M., Ventimiglia M., Cottone M., Orlando A. (2020). Effectiveness and safety of Ustekinumab for the treatment of Crohn’s disease in real-life experiences: A meta-analysis of observational studies. Expert Opin. Biol. Ther..

[78] Rutgeerts P., Gasink C., Chan D., Lang Y., Pollack P., Colombel J.F., Wolf D.C., Jacobstein D., Johanns J., Szapary P. (2018). Efficacy of Ustekinumab for Inducing Endoscopic Healing in Patients with Crohn’s Disease. Gastroenterology.

[79] Bermejo F., Jimenez L., Algaba A., Vela M., Bastida G., Merino O., Lopez-Garcia A., Melcarne L., Rodriguez-Lago I., de la Maza S. (2022). Re-induction with Intravenous Ustekinumab in Patients with Crohn’s Disease and a Loss of Response to This Therapy. Inflamm. Bowel Dis..

[80] Rolston V.S., Kimmel J., Popov V., Bosworth B.P., Hudesman D., Malter L.B., Hong S., Chang S. (2021). Ustekinumab Does Not Increase Risk of Adverse Events: A Meta-Analysis of Randomized Controlled Trials. Dig. Dis. Sci..

[81] Engel T., Yung D.E., Ma C., Pariente B., WIls P., Eliakim R., Ungar B., Ben-Horin S., Kopylov U. (2019). Effectiveness and safety of Ustekinumab for Crohn’s disease; systematic review and pooled analysis of real-world evidence. Dig. Liver Dis..

[82] Honap S., Meade S., Ibraheim H., Irving P.M., Jones M.P., Samaan M.A. (2022). Effectiveness and Safety of Ustekinumab in Inflammatory Bowel Disease: A Systematic Review and Meta-Analysis. Dig. Dis. Sci..

