# Supplementary material for: Real-World Evidence of the Effectiveness and Safety of Ustekinumab for the Treatment of Crohn’s Disease: Systematic Review and Meta-Analysis of Observational Studies
Source: J Clin Med. 2022 Jul 20;11(14):4202. doi: 10.3390/jcm11144202 (PMC9317084; doi:10.3390/jcm11144202)

SUPPLEMENTARY INFORMATION

Supplementary Figure S1: Newcastle-Ottawa quality assessment scale.

NEWCASTLE - OTTAWA QUALITY ASSESSMENT SCALE
COHORT STUDIES

Note: A study can be awarded a maximum of one star for each numbered item within the Selection and Outcome categories. A maximum of two stars can be given for Comparability

Selection

1) Representativeness of the exposed cohort
   a) truly representative of the average _______________ (describe) in the community ✱
   b) somewhat representative of the average ______________ in the community ✱
   c) selected group of users eg nurses, volunteers
   d) no description of the derivation of the cohort

2) Selection of the non exposed cohort
   a) drawn from the same community as the exposed cohort ✱
   b) drawn from a different source
   c) no description of the derivation of the non exposed cohort

3) Ascertainment of exposure
   a) secure record (eg surgical records) ✱
   b) structured interview ✱
   c) written self report
   d) no description

4) Demonstration that outcome of interest was not present at start of study
   a) yes ✱
   b) no

Comparability

1) Comparability of cohorts on the basis of the design or analysis
   a) study controls for _____________ (select the most important factor) ✱
   b) study controls for any additional factor ✱ (This criteria could be modified to indicate specific control for a second important factor.)

Outcome

1) Assessment of outcome
   a) independent blind assessment ✱
   b) record linkage ✱
   c) self report
   d) no description

2) Was follow-up long enough for outcomes to occur
   a) yes (select an adequate follow up period for outcome of interest) ✱
   b) no

3) Adequacy of follow up of cohorts
   a) complete follow up - all subjects accounted for ✱
   b) subjects lost to follow up unlikely to introduce bias - small number lost - > ____ % (select an adequate %) follow up, or description provided of those lost) ✱
   c) follow up rate < ____% (select an adequate %) and no description of those lost
   d) no statement

Supplementary Figure S2. Corticosteroid free-clinical remission.

(A)

Study or Subgroup	SE	Weight	IV, Random, 95% CI
**1.3.1 Global**			
Greenup 2017	0.114	1.2%	0.47 [0.25, 0.69]
Hernández-Camba 2019	0.072	1.8%	0.43 [0.29, 0.57]
Kubesch 2019	0.041	2.4%	0.19 [0.11, 0.27]
Liefferinckx 2019	0.032	2.5%	0.19 [0.13, 0.25]
Townsend 2019	0.08	1.7%	0.20 [0.04, 0.36]
Verstockt 2019	0.05	2.2%	0.31 [0.21, 0.41]
Alric 2020	0.047	2.3%	0.38 [0.29, 0.47]
Biemans 2020	0.035	2.5%	0.24 [0.17, 0.31]
Gadhok 2020	0.034	2.5%	0.40 [0.33, 0.47]
Sánchez-Rodríguez 2020	0.085	1.6%	0.24 [0.07, 0.41]
Viola 2021	0.042	2.4%	0.35 [0.27, 0.43]
Gonczi 2021	0.044	2.3%	0.44 [0.35, 0.53]
Lorenzo 2021	0.051	2.2%	0.32 [0.22, 0.42]
Manlay 2021	0.035	2.5%	0.56 [0.49, 0.63]
Straatmijer 2021	0.029	2.6%	0.32 [0.26, 0.38]
**Subtotal (95% CI)**		**32.9%**	**0.33 [0.27, 0.40]**

Heterogeneity: Tau² = 0.01; Chi² = 99.91, df = 14 (P < 0.00001); I² = 86%
Test for overall effect: Z = 10.79 (P < 0.00001)

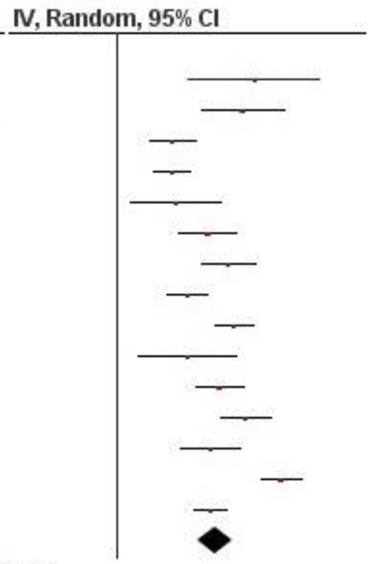

(B)

Study or Subgroup	SE	Weight	IV, Random, 95% CI
**2.3.1 Global**			
Battat 2017	0.064	1.1%	0.50 [0.37, 0.63]
Hoffmann 2019	0.063	1.1%	0.35 [0.23, 0.47]
Liefferinckx 2019	0.036	1.2%	0.27 [0.20, 0.34]
Verstockt 2019	0.052	1.1%	0.38 [0.28, 0.48]
Townsend 2019	0.096	0.9%	0.36 [0.17, 0.55]
Kakkadasam 2020	0.057	1.1%	0.49 [0.38, 0.60]
Tomasic 2020	0.075	1.0%	0.38 [0.23, 0.53]
Saldaña 2020	0.084	1.0%	0.46 [0.30, 0.62]
Bar-Gil Shitrit 2020	0.051	1.1%	0.11 [0.01, 0.21]
Biemans 2020	0.039	1.2%	0.38 [0.30, 0.46]
Casas 2020	0.031	1.2%	0.07 [0.01, 0.13]
Gadhok 2020	0.031	1.2%	0.28 [0.22, 0.34]
Gubbiotti 2020	0.049	1.1%	0.51 [0.41, 0.61]
Sánchez-Rodríguez 2020	0.092	0.9%	0.29 [0.11, 0.47]
Lorenzo 2021	0.064	1.1%	0.44 [0.31, 0.57]
Bokemeyer 2021	0.027	1.2%	0.53 [0.48, 0.58]
Garg 2021 (a)	0.102	0.9%	0.30 [0.10, 0.50]
Garg 2021 (b)	0.082	1.0%	0.54 [0.38, 0.70]
Manlay 2021	0.038	1.2%	0.62 [0.55, 0.69]
Scribano 2021	0.041	1.2%	0.61 [0.53, 0.69]
Straatmijer 2021	0.031	1.2%	0.41 [0.35, 0.47]
Tursi 2021	0.035	1.2%	0.60 [0.53, 0.67]
Gonczi 2021	0.043	1.2%	0.58 [0.50, 0.66]
Chaparro 2022	0.023	1.2%	0.53 [0.48, 0.58]
**Subtotal (95% CI)**		**26.3%**	**0.42 [0.35, 0.49]**

Heterogeneity: Tau² = 0.03; Chi² = 346.38, df = 23 (P < 0.00001); I² = 93%
Test for overall effect: Z = 12.03 (P < 0.00001)

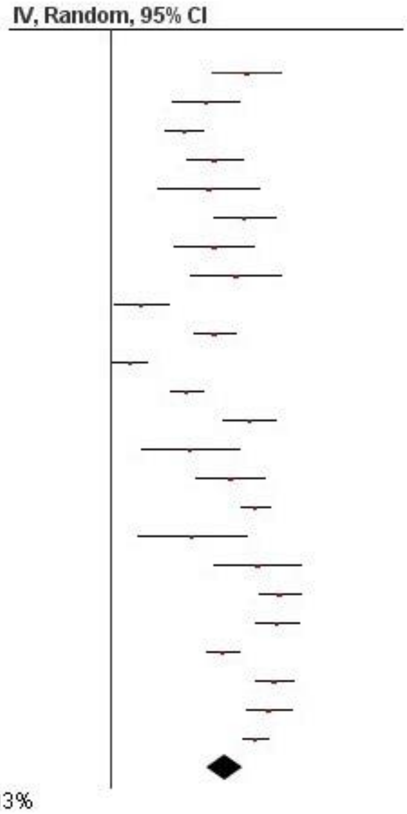

(C)

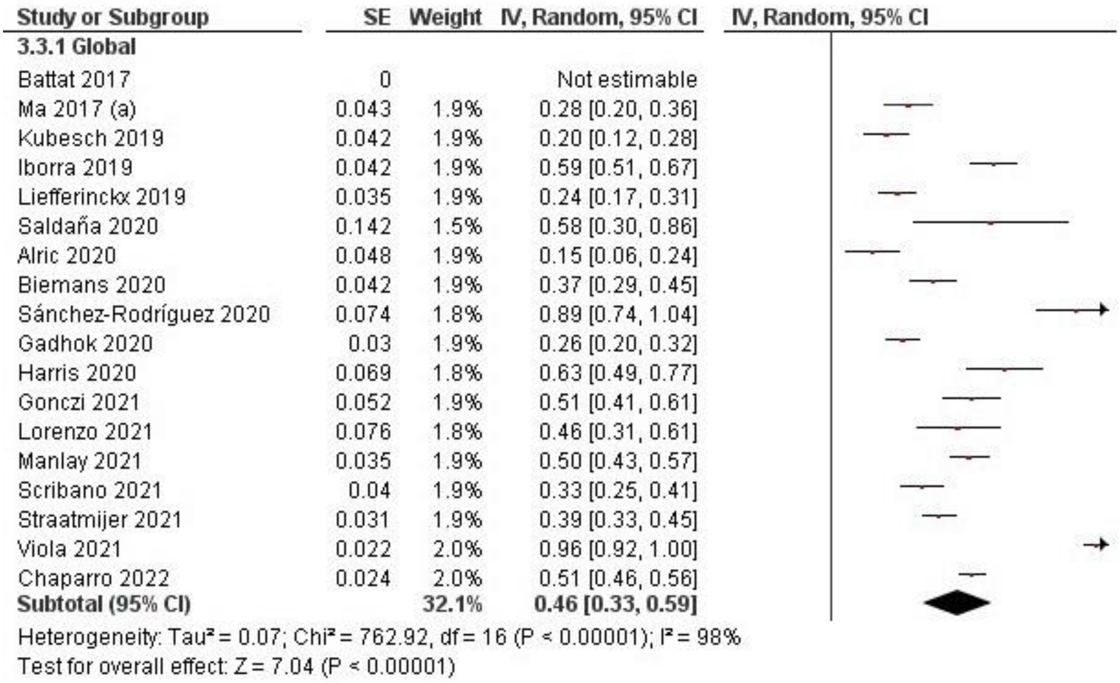

(A) Corticosteroid free clinical remission in short-term (8w-14w), (B) Corticosteroid free clinical remission in medium-term (16w-24w), (C) Corticosteroid free clinical remission in long-term (48w-52w)

Supplementary Figure S3. Endoscopic remission.

(A)

Study or Subgroup	SE	Weight	IV, Random, 95% CI
**4.1.1 Global**			
Kopylov 2014	0.1	0.5%	0.15 [-0.05, 0.35]
Battat 2017	0.053	1.7%	0.20 [0.10, 0.30]
Verstockt 2019	0.028	4.4%	0.07 [0.02, 0.12]
Hoffmann 2019	0.001	12.2%	0.01 [0.01, 0.01]
Lynn 2019	0.154	0.2%	0.17 [-0.13, 0.47]
Truyens 2020	0.061	1.3%	0.06 [-0.06, 0.18]
af Björkesten 2020	0.116	0.4%	0.35 [0.12, 0.58]
Bennett 2020	0.065	1.2%	0.69 [0.56, 0.82]
Garg 2021 (a)	0.052	1.7%	0.25 [0.15, 0.35]
Garg 2021 (b)	0.054	1.6%	0.30 [0.19, 0.41]
Plevris 2021	0.037	3.0%	0.10 [0.03, 0.17]
Tursi 2021	0.063	1.2%	0.53 [0.41, 0.65]
**Subtotal (95% CI)**		**29.3%**	**0.24 [0.13, 0.34]**

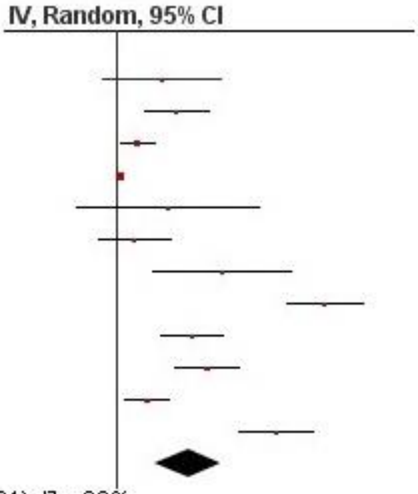

Heterogeneity: $Tau^2 = 0.03$; $Chi^2 = 262.82$, df = 11 ($P < 0.00001$); $I^2 = 96\%$
Test for overall effect: Z = 4.31 ($P < 0.0001$)

(B)

Study or Subgroup	SE	Weight	IV, Random, 95% CI
**4.2.1 Global**			
Ma 2017 (a)	0.046	3.7%	0.27 [0.18, 0.36]
Ma 2017 (b)	0.051	3.5%	0.41 [0.31, 0.51]
Hoffmann 2019	0.119	1.8%	0.41 [0.18, 0.64]
Iborra 2019	0.029	4.1%	0.16 [0.10, 0.22]
Lynn 2019	0.019	4.3%	0.34 [0.30, 0.38]
Truyens 2020	0.083	2.6%	0.13 [-0.03, 0.29]
Calvo 2020	0.09	2.4%	0.64 [0.46, 0.82]
af Björkesten 2020	0.111	2.0%	0.33 [0.11, 0.55]
Shim 2020	0.219	0.8%	0.40 [-0.03, 0.83]
Miranda 2021	0.055	3.4%	0.35 [0.24, 0.46]
Plevris 2021	0.107	2.1%	0.32 [0.11, 0.53]
**Subtotal (95% CI)**		**30.6%**	**0.33 [0.25, 0.40]**

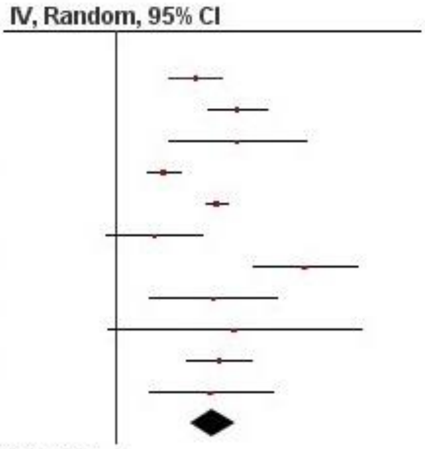

Heterogeneity: $Tau^2 = 0.01$; $Chi^2 = 53.21$, df = 10 ($P < 0.00001$); $I^2 = 81\%$
Test for overall effect: Z = 8.35 ($P < 0.00001$)

(A) Endoscopic remission in medium-term (16w-24w), (B) Endoscopic remission in long-term (48w-52w).

Supplementary Figure S4: Publication bias.

(A) Clinical response short-term

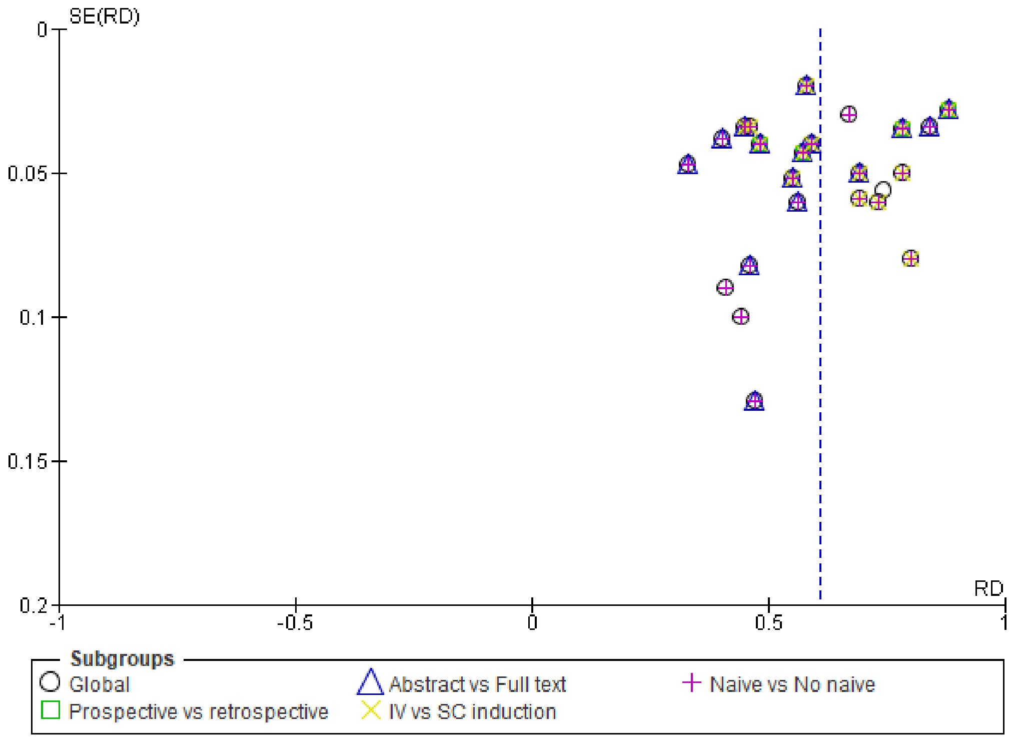

(B) **Clinical remission short-term**

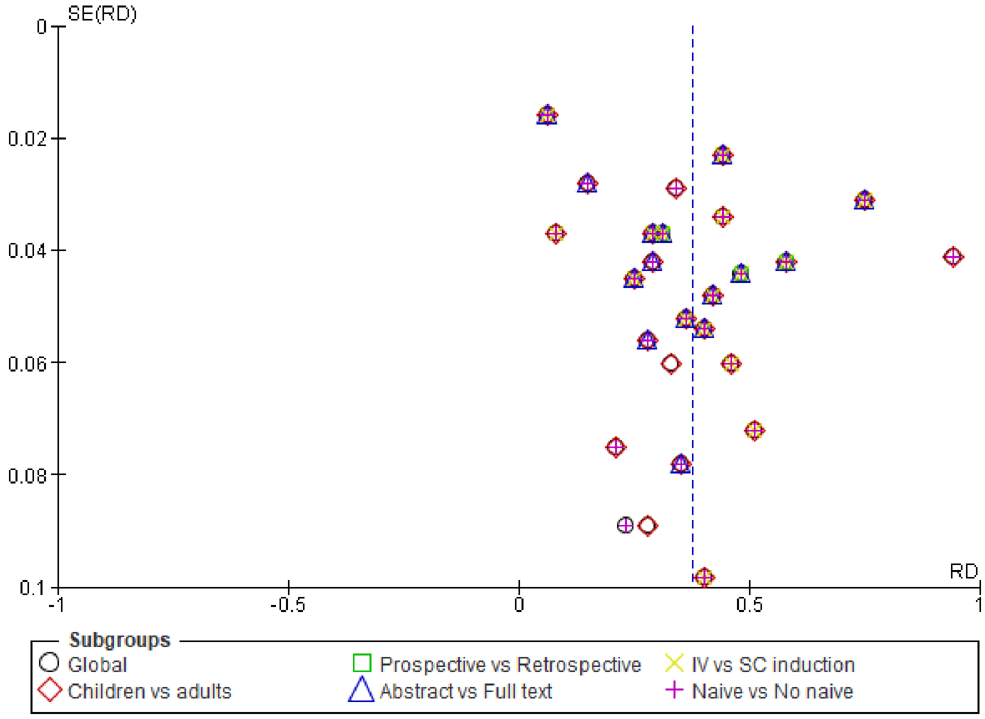

(C) Corticosteroid-free clinical remission short-term

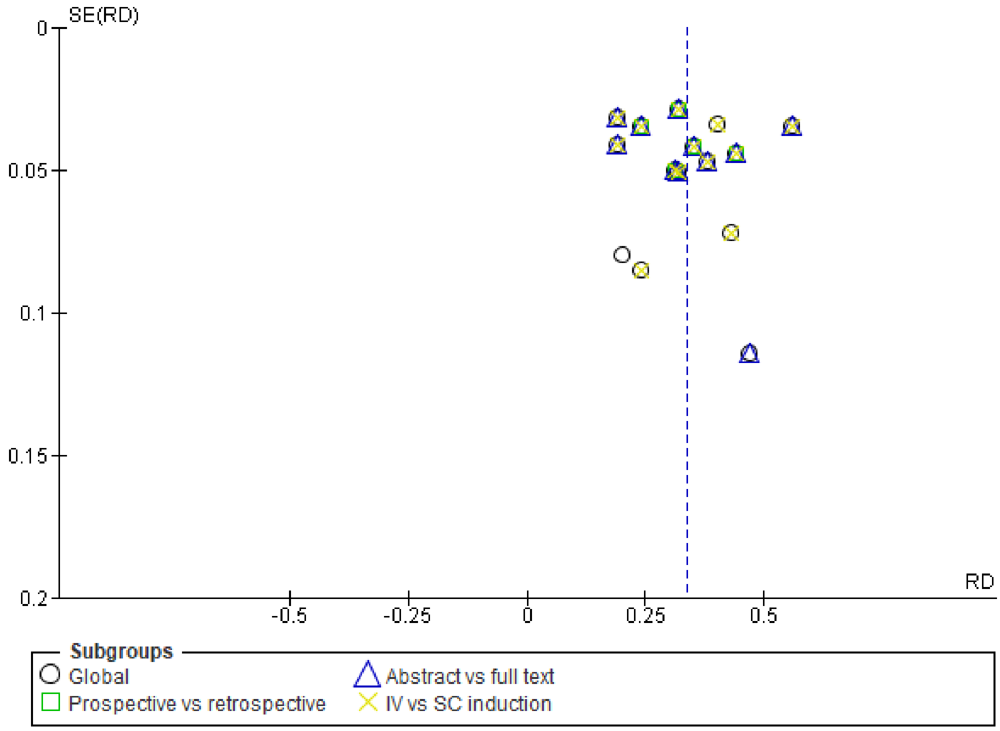

(D) Clinical response medium-term

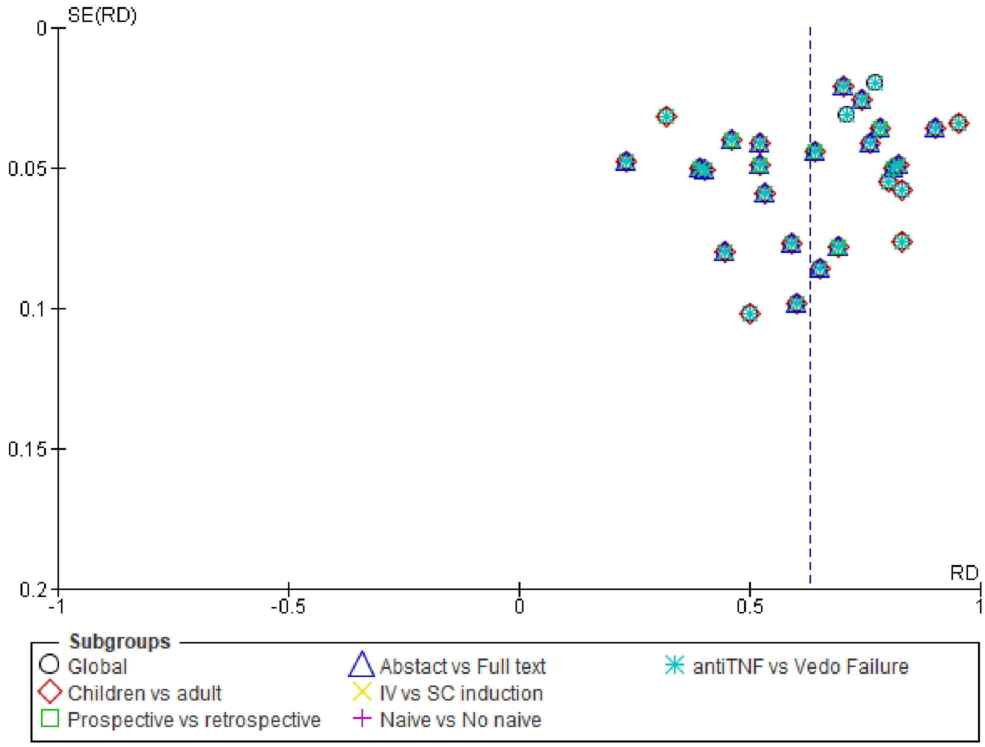

(E) Clinical remission medium-term

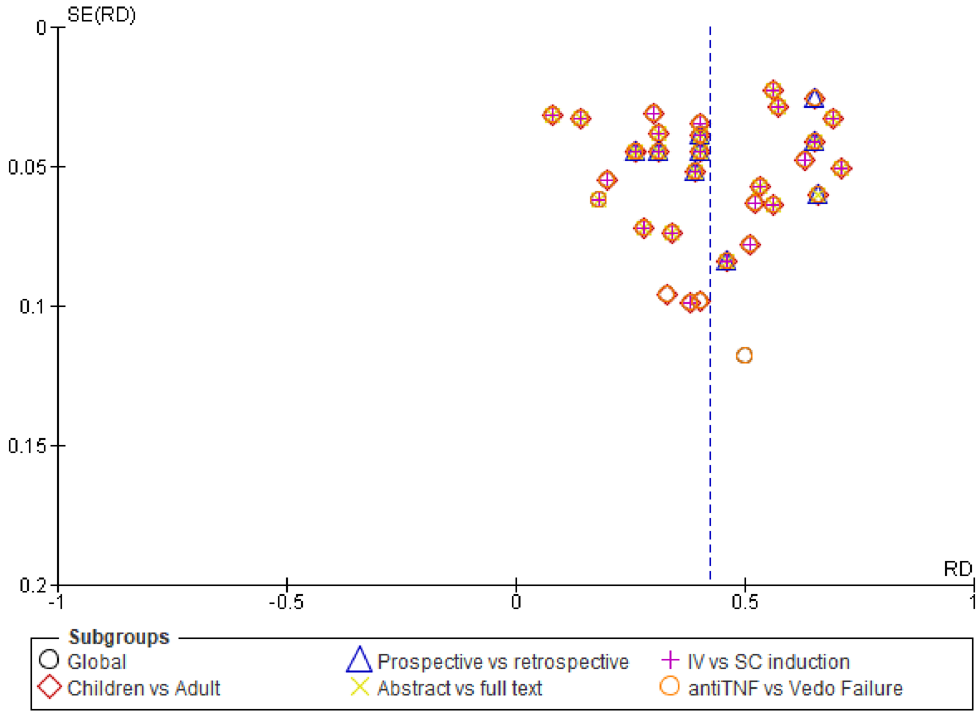

(F) Corticosteroid-free clinical remission medium-term

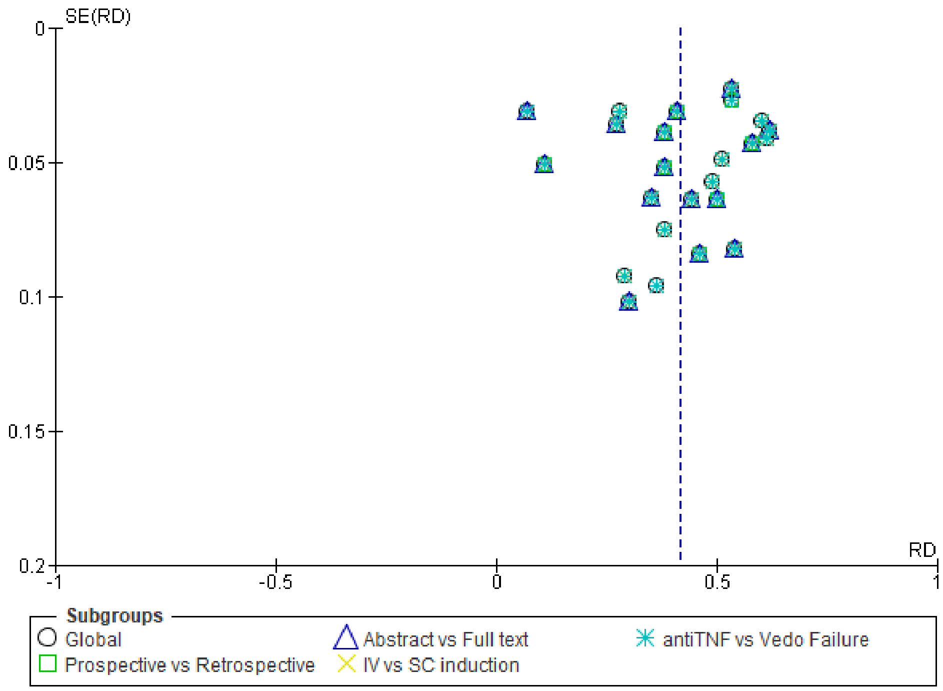

(G) Endoscopic remission medium-term

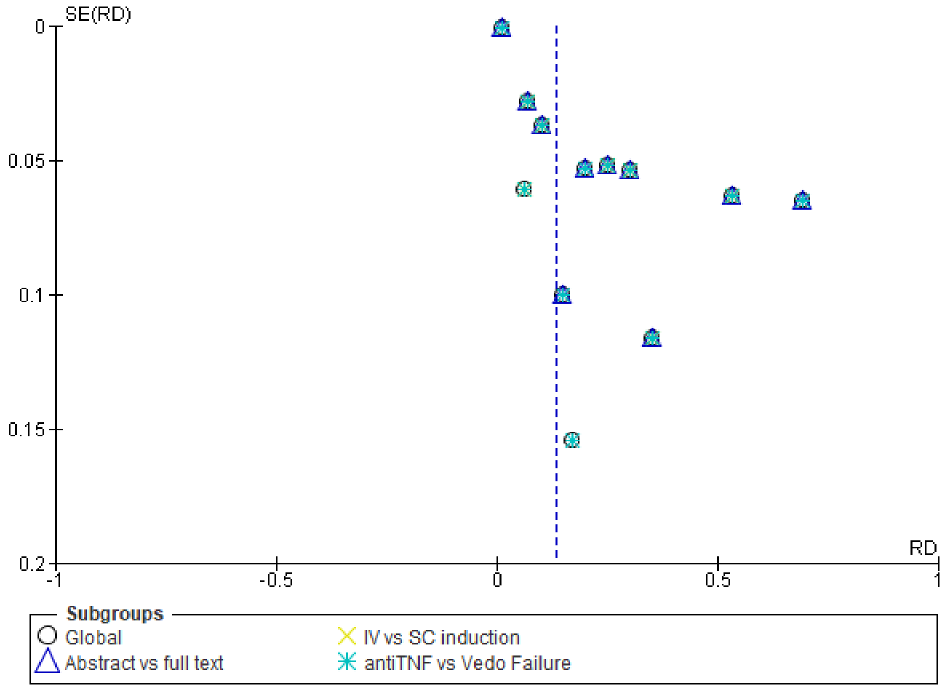

(H) Clinical response long-term

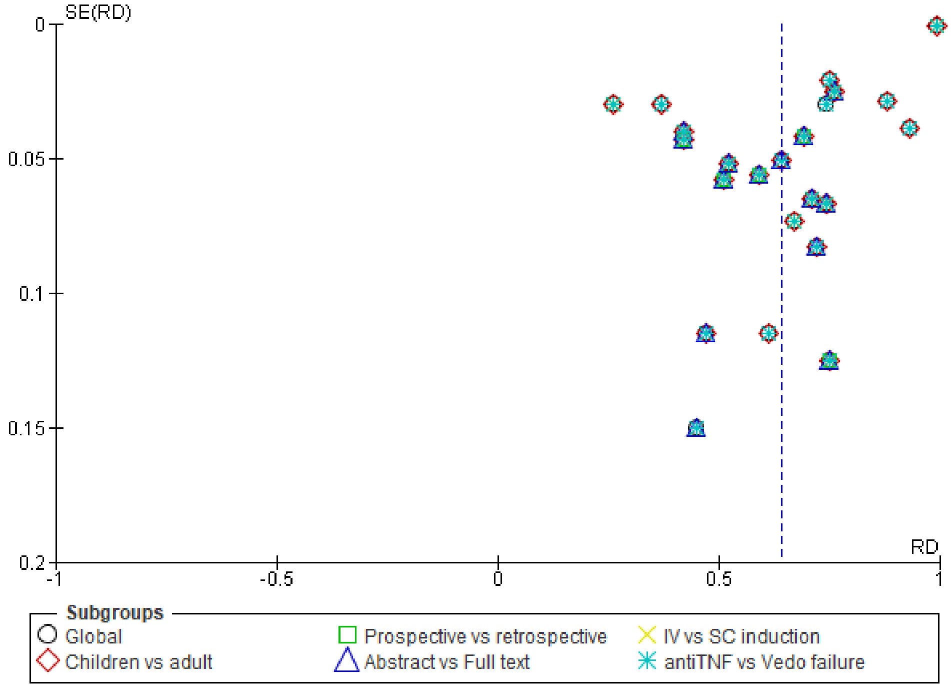

(I) Clinical remission long-term

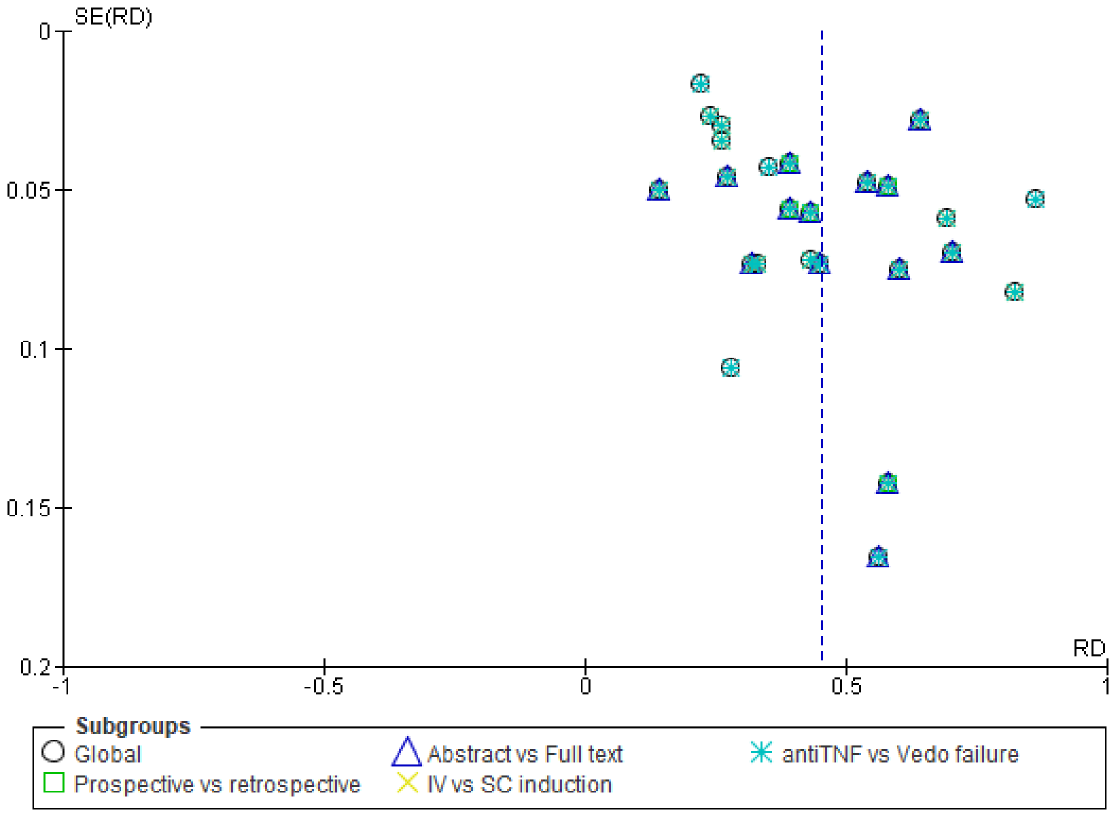

(J) Corticosteroid-free clinical remission long-term

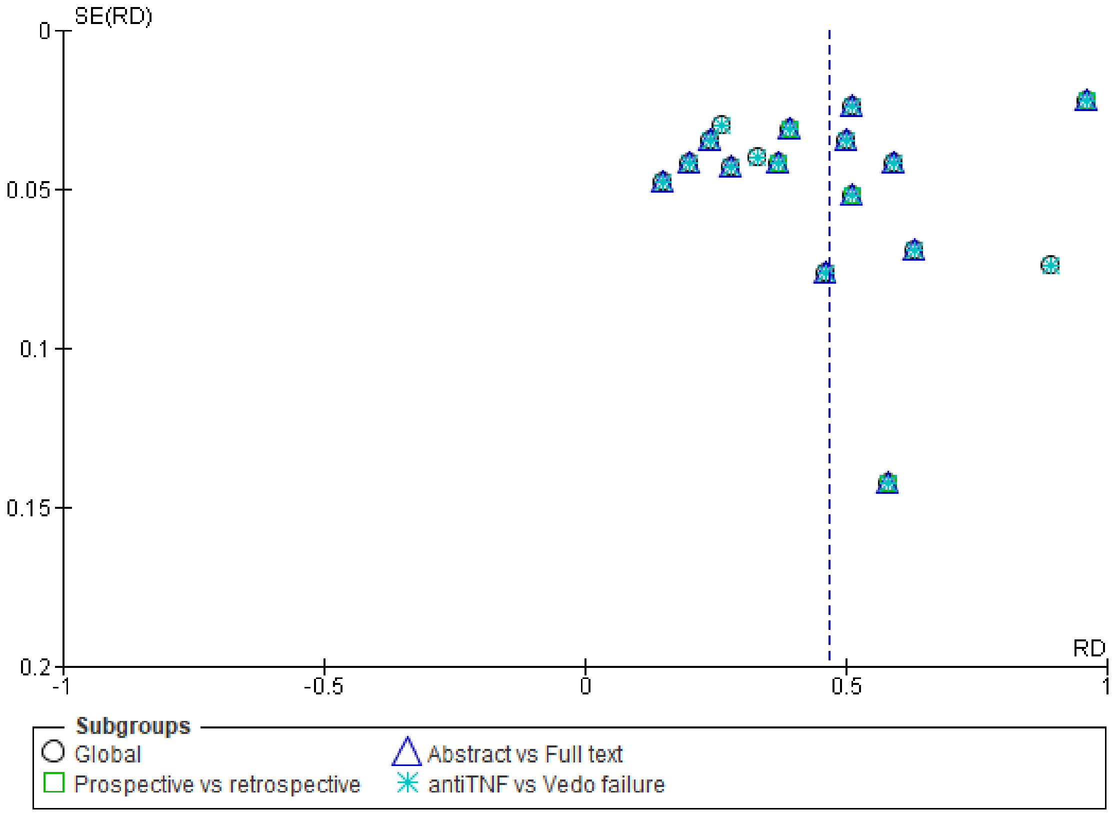

(K) Endoscopic remission long-term

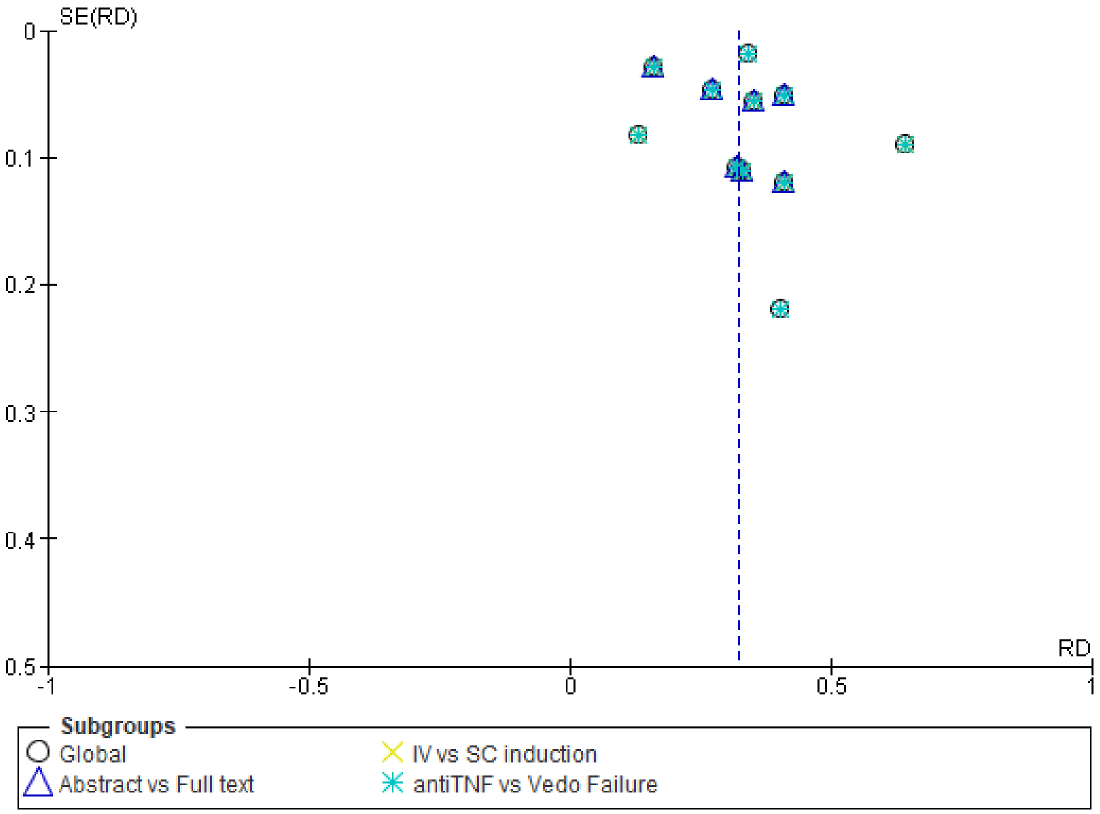

Supplement: Supplementary file 1 [file jcm-11-04202-s001.zip › jcm-1806163-supplementary.pdf]
